# Comprehensive Study of Artificial Light‐Harvesting Systems with a Multi‐Step Sequential Energy Transfer Mechanism

**DOI:** 10.1002/advs.202404269

**Published:** 2024-06-14

**Authors:** Yong Wu, Yuqian Wang, Xu Yu, Qiao Song

**Affiliations:** ^1^ Shenzhen Grubbs Institute Southern University of Science and Technology Shenzhen 518055 China; ^2^ Institute of Innovation Materials and Energy College of Chemistry and Chemical Engineering Yangzhou University Yangzhou 225002 China; ^3^ Guangming Advanced Research Institute Southern University of Science and Technology Shenzhen 518055 China

**Keywords:** artificial light‐harvesting systems, cyclic peptides, sequential energy transfer, supramolecular scaffolds

## Abstract

Artificial light‐harvesting systems (LHSs) with a multi‐step sequential energy transfer mechanism significantly enhance light energy utilization. Nonetheless, most of these systems exhibit an overall energy transfer efficiency below 80%. Moreover, due to challenges in molecularly aligning multiple donor/acceptor chromophores, systems featuring ≥3‐step sequential energy transfer are rarely reported. Here, a series of artificial LHSs is introduced featuring up to 4‐step energy transfer mechanism, constructed using a cyclic peptide‐based supramolecular scaffold. These LHSs showed remarkably high energy transfer efficiencies (≥90%) and satisfactory fluorescence quantum yields (ranging from 17.6% to 58.4%). Furthermore, the structural robustness of the supramolecular scaffold enables a comprehensive study of these systems, elucidating the associated energy transfer pathways, and identifying additional energy transfer processes beyond the targeted sequential energy transfer. Overall, this comprehensive investigation not only enhances the understanding of these LHSs, but also underscores the versatility of cyclic peptide‐based supramolecular scaffolds in advancing energy harvesting technologies.

## Graphical abstract

A series of artificial light‐harvesting systems is developed utilizing a cyclic peptide‐based supramolecular scaffold, showcasing an up to 4‐step energy transfer mechanism and demonstrating exceptional energy transfer efficiencies of ≥90%. Through comprehensive analysis, the associated energy transfer pathways inherent in these systems are uncovered, along with the supplementary energy transfer processes that extend beyond the initial targeted sequential transfers.

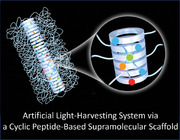

## 1 Introduction

The light‐harvesting systems (LHSs) found in nature are precise supramolecular assemblies of chromophores, adept at efficiently harvesting light energy through a multi‐step sequential energy transfer process.^[^ ^1^ ^]^ Inspired by nature, scientists have increasingly focused on fabricating cascade LHSs using assorted supramolecular scaffolds in recent years, including host‐guest assemblies,^[^ ^2^ ^]^ metallacycles/stacks,^[^ ^3^ ^]^ biomacromolecules,^[^ ^4^ ^]^ and supramolecular polymers.^[^ ^5^ ^]^ However, a large majority of the examples exhibit an overall energy transfer efficiency lower than 80%.^[^ ^6^ ^]^ The key to enhancing the efficiency lies in arranging a series of donor and acceptor dyes in close proximity within specific supramolecular scaffolds, facilitating efficient Förster resonance energy transfer (FRET) between them. Meanwhile, the scope of most artificial sequential LHSs remains confined to preliminary spectral analysis. Deeper investigations could furnish additional insights into their performance and offer invaluable guidance for their refinement. Unfortunately, such endeavors are frequently hindered by the intricate nature of supramolecular systems, rendering them challenging or even unfeasible.^[^ ^7^ ^]^ In addition, while artificial sequential LHSs with 2‐step sequential energy transfer processes are frequently reported, those with three or more steps are seldom encountered in the literature.^[^ ^8^ ^]^

Self‐assembling cyclic peptides are capable of stacking into nanotubular structures through multiple hydrogen bonding interactions.^[^ ^9^ ^]^ When a hydrophilic polymer chain is attached to the cyclic peptide, it results in the creation of a well‐defined core‐shell cylindrical structure.^[^ ^10^ ^]^ This precisely organized structure has been utilized as a supramolecular scaffold to molecularly align functional elements, including drugs, *π*‐conjugated chromophores, proteins, and even nanoparticles.^[^ ^11^ ^]^ Particularly, various photo‐functional supramolecular materials have been constructed, including ultrabright fluorescence nanoparticles, thermally activated delayed fluorescence materials, and artificial LHSs.^[^ ^12^ ^]^ This implies the powerful capability of the supramolecular scaffold in aligning *π*‐conjugated chromophores at the molecular level. Therefore, we envision that the supramolecular scaffold would facilitate the arrangement of a series of donor and acceptor dyes in close proximity, enabling the fabrication of artificial LHSs with three or more steps. Most importantly, given the fact that the self‐assembled structure of the supramolecular scaffold is unlikely to be affected by the properties of dye moieties, it offers an ideal system for a comprehensive study of the energy transfer processes of the artificial LHSs.

**Scheme 1:**
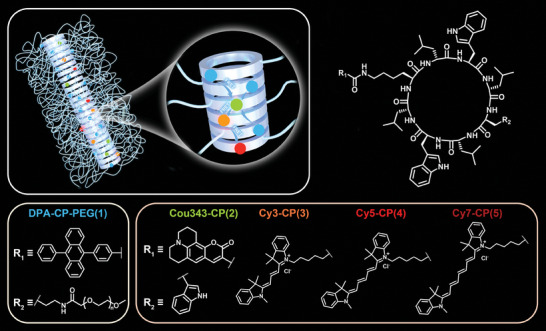
Cartoon illustration demonstrating the construction of an artificial light‐harvesting system with a multi‐step sequential energy transfer mechanism.

In this work, we report artificial LHSs with a multi‐step sequential FRET process constructed from a cyclic peptide‐based supramolecular scaffold in water. As shown in **Scheme** **1**, five fluorophores are rationally selected and conjugated onto the cyclic peptide to obtain the corresponding fluorophore‐cyclic peptide‐polymer conjugate (marked as DPA‐CP‐PEG) or fluorophore‐cyclic peptide conjugates (marked as Cou343‐CP, Cy3‐CP, Cy5‐CP, and Cy7‐CP). By co‐assembling the building blocks, the fluorophores are brought in close proximity and molecularly aligned along the nanotubular structures. The good spectral overlap between the absorption spectra of the acceptors and the emission spectra of the donors at each level guarantees the efficiency of each FRET process, yielding artificial LHSs with up to a 4‐step sequential energy transfer process. The performance of the artificial LHSs was comprehensively assessed, revealing high energy transfer efficiencies (>90%) and satisfactory fluorescence quantum yields (17.6–58.4%). Moreover, a thorough study into these artificial LHSs elucidated the associated energy transfer pathways and pointed out the presence of additional energy transfer processes beyond the targeted sequential energy transfer. This comprehensive analysis underscores the potential of cyclic peptide‐based supramolecular scaffolds as a powerful platform for developing high‐performance cascade LHSs.

## 2 Results and Discussion

### 2.1 Synthesis and Self‐Assembly

9,10‐Diphenylanthracene (DPA) is selected as the energy donor due to its high fluorescence quantum yield and relatively narrow emission spectrum. The fluorophore‐cyclic peptide‐polymer conjugate, DPA‐CP‐PEG, was synthesized using an orthogonal protecting group chemistry using a cyclic peptide possessing both Boc‐ and Dde‐ protective groups. After the elimination of the Boc groups with trifluoroacetic acid, the DPA moiety was first attached to the cyclic peptide via amide coupling chemistry (Figure S1, Supporting Information). Deprotection of the Dde group using hydrazine was performed to afford a further reactive lysine group, which was subsequently used to attach the hydrophilic polymer, poly(ethylene glycol), to obtain DPA‐CP‐PEG using the same amide coupling method. The successful synthesis of DPA‐CP‐PEG was proved by LC‐MS, MALDI‐TOF MS, and ^1^H NMR (Figure S2, Supporting Information). The fluorophore‐cyclic peptide conjugates (Cou343‐CP, Cy3‐CP, Cy5‐CP, and Cy7‐CP) were synthesized by reacting the corresponding fluorophores with cyclic peptide via amide coupling chemistry.

**Figure 1:**
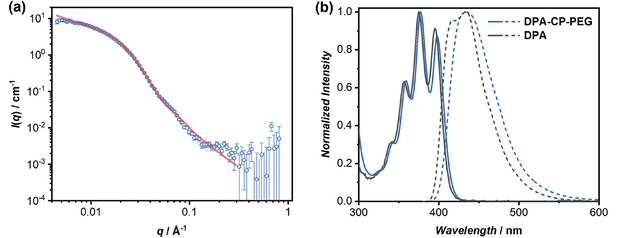
Self‐assembly and photophysical properties of DPA‐CP‐PEG. a) SANS data and fitting to a cylindrical micelle model of DPA‐CP‐PEG; b) Normalized absorption spectra (solid curves) and fluorescence spectra (dashed curves) of DPA‐CP‐PEG and DPA.

Multiple hydrogen bonding interactions between the cyclic peptides are the driving forces of forming self‐assembled core‐shell cylindrical structures. The self‐assembling behavior of DPA‐CP‐PEG was investigated by small angle neutron scattering (SANS). **Figure** **1**a shows the reduced, corrected SANS data of DPA‐CP‐PEG in deuterated water. Using SASfit software, the data could be fitted to a cylindrical micelle model, supporting the formation of the core‐shell cylindrical structures. The average length and diameter were determined to be 14.2 and 8.3 nm, respectively (Table S1, Supporting Information). Furthermore, the self‐assembly of DPA‐CP‐PEG in water was revealed by the change in photophysical properties. As shown in Figure 1b, a slight bathochromic shift was observed in both the absorption and fluorescence spectra of DPA‐CP‐PEG, compared to free DPA. Meanwhile, the fluorescence quantum yield (*Φ*_F_) of DPA‐CP‐PEG in water was determined to be 40.1%, guaranteeing its employment as the energy donor for the construction of artificial LHSs.

### 2.2 Binary LHS with One‐Step Energy Transfer

**Figure 2:**
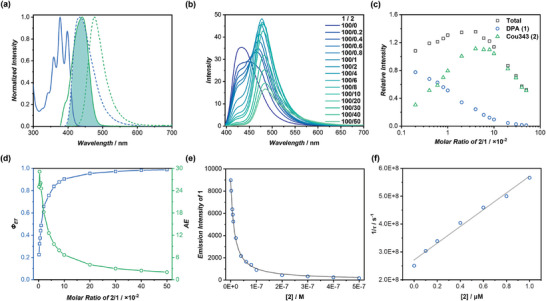
Energy transfer behaviors of the binary system of **1**/**2**. a) Normalized fluorescence spectra (dashed curves) of DPA‐CP‐PEG (**1**, blue trace) and Cou343‐CP (**2**, green trace), and their normalized absorption spectra (solid curves); b) Fluorescence spectra of DPA‐CP‐PEG with different concentrations of Cou343‐CP (*λ*_ex_ = 377 nm); c) Ratiometric plot of the total, DPA, and Cou343 emission intensities; d) *Φ*_ET_ and *AE* values at different **2**/**1** molar ratios; e) Non‐linear fitting of the emission intensities of **1** versus the concentration of **2**; f) Plots of 1/*τ* values of **1** versus the concentration of **2**.

We initially explored the FRET process between DPA‐CP‐PEG (**1**) and Cou343‐CP (**2**). As shown in **Figure** **2**a, the emission spectrum of **1** exhibited significant overlap with the absorption spectrum of **2**. Consequently, we anticipated an efficient energy transfer process upon co‐assembling **1** and **2**. Indeed, as indicated in Figure 2b, with an increasing **2**/**1** molar ratio, the DPA emission intensity, peaking at 432 nm, gradually attenuated. Simultaneously, a new emission band at 483 nm ascribed to Cou343 emerged when excited at 377 nm. The occurrence of FRET between **1** and **2** was further corroborated by time‐resolved fluorescence spectroscopy (TRFS), revealing a decrease from 4.0 ns for **1** alone to 1.8 ns with the addition of 10% of **2** (Figure S3, Supporting Information).

For a precise comprehension of the FRET process between **1** and **2**, the emission spectra were deconvoluted into two peaks corresponding to DPA and Cou343 (Figure S4, Supporting Information). This approach allowed for a quantitative analysis of the emissions from both DPA and Cou343 (Figure S5, Supporting Information). Initially, the ratiometric plot of **2**/**1 (*I*_2_/*I*_1_)** exhibited a linear increase with the molar ratio of **2**/**1**, suggesting a uniform distribution of acceptor **2** within the assemblies formed by **1** (Figure S6, Supporting Information), while the self‐assembled core‐shell cylindrical structures were maintained during the co‐assembling process.^[^ ^12a,b^ ^]^ Subsequently, the evolution of the emission intensities of DPA and Cou343 was investigated concerning the molar ratio of **2**/**1**. As illustrated in Figure 2c, the emission intensity of DPA consistently decreased with the increase of **2**/**1** molar ratio, aligning with the FRET mechanism. Regarding Cou343, a rapid intensity increase was witnessed as the **1**/**2** molar ratio elevated from 100/0 to 100/4. However, with a further increase in **2** concentration (from 100/4 to 100/50), a gradual decrease in intensity occurred. This phenomenon was likely attributed to the aggregation‐caused quenching (ACQ) of Cou343 at higher concentrations, as revealed by a gradual bathochromic shift of Cou343 emission as the concentration increased (Figure S7, Supporting Information). Consequently, the overall emission intensity exhibited an initial increase from 100/0 to 100/4, followed by a subsequent decline 100/4 to 100/50.

To access the performance of the LHS composed of **1** and **2**, key parameters including energy transfer efficiency (*Φ*_ET_), antenna effect (*AE*), and *Φ_F_* were measured and calculated (Table S2, Supporting Information). As depicted in Figure 2d, *Φ*_ET_ values exhibited an upward trend with the increasing **2**/**1** molar ratio, surpassing 90% when the **1**/**2** molar ratio reached 100/10 and eventually nearing 99% at a molar ratio of 100/50. The *AE* values, however, demonstrated a declining trend with the rise in the **2**/**1** molar ratio, ranging from a high of 29 to 2. For instance, at a **1**/**2** molar ratio of 100/8, *Φ*_ET_ was calculated to be 87.8% with an *AE* value of 7.9, whereas, at a **1**/**2** molar ratio of 100/10, *Φ*_ET_ was 90.5% with an *AE* value of 6.7. Markedly, **1** exhibited a notable *Φ_F_* value of 40.1%, which further increased to as high as 58.4% at a **1**/**2** molar ratio of 100/10. Therefore, a high‐performance artificial LHS was developed, showcasing exceptional energy transfer efficiency and luminescent capability.

It is worth noting the significant differences between Stern–Volmer plots of the average fluorescence lifetime of **1** and the steady‐state fluorescence intensity of **1** (Figure S8, Supporting Information). These trends suggest that in the light‐harvesting system comprising **1** and **2**, both static and dynamic quenching contribute to the fluorescent emission quenching of **1**. Dynamic quenching involves direct hetero‐energy transfer between the donor/acceptor pairs, while static quenching requires homo‐energy transfer within donors before transferring energy to the acceptor. Since **1** serves as the donor matrix with a densely packed character, donor–donor energy migration, also known as homo‐FRET, is involved. As indicated in Table S3 (Supporting Information), the percentage of dynamic quenching markedly increased with the increase of **2**/**1** molar ratio, ranging from 17.5% to as high as 71.9%. Meanwhile, the percentage of static quenching remained relatively constant in the range of 30–40%, irrespective of the changes in the **2**/**1** molar ratio. This behavior could be attributed to the decrease in the average distance between **2** and **1** with the increase of **2**, while no change in the average distance between **1** is expected since the majority of the system remains comprised of **1**. Therefore, dynamic quenching is more likely to occur, whereas static quenching remains at a constant level with the increase of **2**/**1** molar ratio. To determine the number of donors (*n*) that can be quenched by a single acceptor, a mathematical model combining both dynamic and static quenching mechanisms was employed. Through non‐linear fitting of the emission intensities of **1** versus the concentration of **2**, the calculated *n* value reached as high as 252 (Figure 2e). In addition, by plotting the 1/τ values of **1** versus the concentration of **2**, the second‐order exciton migration rate constant of **1**/**2** is determined to be 3.0 × 10^14^ L mol^−1^ s^−1^ (Figure 2f). It is much larger than the diffusion limit for the bimolecular reaction in solution and on par with the highest values reported.^[^ ^3^, ^13^ ^]^ Overall, this underscores the remarkable advantage of utilizing the cyclic peptide‐based supramolecular scaffold in artificial light‐harvesting systems.

### 2.3 Ternary LHS with Two‐Step Sequential Energy Transfer

**Figure 3:**
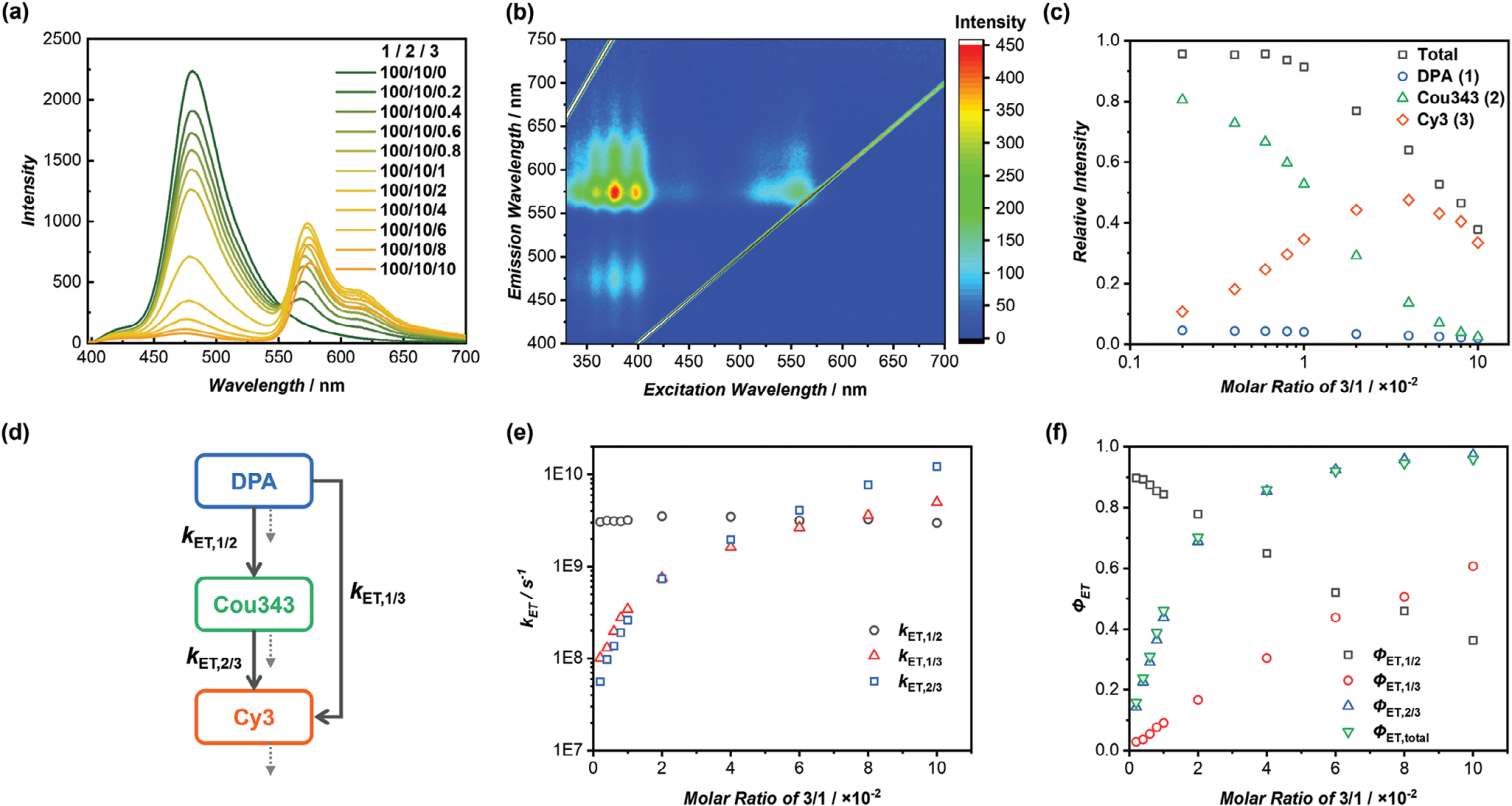
Energy transfer behaviors of the ternary system of **1**/**2**/**3**. a) Fluorescence spectra of DPA‐CP‐PEG/Cou343‐CP = 100/10 with different concentrations of Cy3‐CP (*λ*_ex_ = 377 nm); b) 2D excitation spectra of **1**/**2**/**3** (100/10/8); c) Ratiometric plot of the total, DPA, Cou343, and Cy3 emission intensities; d) Energy transfer processes of the **1**/**2**/**3** ternary system; e) Evolution of *k*_ET, 1/2_, *k*_ET, 1/3_, and *k*_ET, 2/3_ of the **1**/**2**/**3** system with the increase of **3** molar ratios; f) Evolution of *Φ*_ET, 1/2_, *Φ*_ET, 1/3_, *Φ*_ET, 2/3_ and *Φ*_ET, total_ with the increase of **3** molar ratios.

Nature harnesses multi‐step sequential energy transfer mechanisms rather than relying on a single‐step process, facilitating more effective light utilization across a wider wavelength spectrum. We investigated the feasibility of constructing a multi‐step sequential energy transfer LHS based on the cyclic peptide‐based supramolecular scaffold. Cy3‐CP (**3**) was strategically selected as the second acceptor to capture light emitted from **2**, given that the absorption spectrum of **3** overlaps well with the emission spectrum of **2** (Figure S9, Supporting Information). To facilitate the envisioned sequential energy transfer process, we created a three‐component system by co‐assembling **1**, **2**, and **3** in various molar ratios. Specifically, a small amount of **3** was incorporated into **1**/**2** (100/10). As shown in **Figure** **3**a, upon excitation at 377 nm, the emission of **2** centered at 480 nm diminished, accompanied by the concurrent enhancement of a new emission band peaking at 573 nm attributed to **3**, leading to an apparent Stokes shift of 196 nm. These data indicate the occurrence of a 2‐step sequential FRET process, which is supported by TRFS experiments (Figure S10, Supporting Information). The sequential energy transfer phenomena are further substantiated by comparing the 2D excitation spectra of **1**/**2** and **1**/**2**/**3** (Figure 3b; Figure S11, Supporting Information). With excitation in the range of 350–450 nm, the emission signal of **2** at 450–550 nm exhibited substantial reduction upon the incorporation of **3**. Simultaneously, an intense emission band at 550–650 nm became evident for **1**/**2**/**3**. Moreover, the emission band of **3** was observed upon excitation across a broad spectrum ranging from 350 to 575 nm, highlighting the advantages of LHSs with multi‐step sequential energy transfer mechanisms.

To gain a precise understanding of the ternary system of **1**/**2**/**3**, the emission spectra were meticulously deconvoluted into 3 peaks corresponding to DPA, Cou343, and Cy3 (Figures S12 and S13, Supporting Information). Similar to the **1**/**2** system, the ratiometric plot of ***I*_3_/(*I*_1_+*I*_2_)** exhibited a linear increase with the molar ratio of **3**/**1**, indicating even distribution of acceptor **3** within the assemblies formed by **1*/*2** (Figure S14, Supporting Information). Next, we examined the evolution of the emission intensities of DPA, Cou343, and Cy3 upon the increase of the molar ratio of **3**/**1**. As shown in Figure 3c, both DPA and Cou343 displayed a continuous decrease in intensity with the addition of **3**, albeit at distinctive rates. Regarding Cy3, a rapid increase in intensity was witnessed when the **3**/**1** molar ratio increased from 0% to 4%, followed by a gradual decrease when the **3**/**1** molar ratio exceeded 4% due to ACQ. The performance of the three‐component LHS composed of **1**/**2/3** was then evaluated (Table S4, Supporting Information). As illustrated in Figure S15 (Supporting Information), *Φ*_ET_ values between **2**/**3** increased with the rise of **3**/**1** molar ratio, surpassing 90% when the **1/2/3** molar ratio reached 100/10/6, and eventually reaching as high as 97.3% at 100/10/10. The *AE* values, however, demonstrated a decreasing trend with the increase of **3**/**1** molar ratio, ranging from 8.0 to 2.4. To our delight, the *Φ*_F_ value remained at a high level of 28.7% at a 1/2/3 molar ratio of 100/10/6. Overall, this LHS exhibited superior performance compared to other reported ternary systems in aqueous media (Table S5 and Figure S16, Supporting Information).

In the ternary system of **1**/**2**/**3**, both static and dynamic quenching exist in the fluorescent emission quenching of **2**, as revealed by Stern–Volmer plots in Figure S17 (Supporting Information). As shown in Table S6 (Supporting Information), the percentage of dynamic quenching increased notably along with the increase of **3** molar ratio, ranging from 10.4% to as high as 82.9%. Meanwhile, the percentage of static quenching remained within the range of 10–30%, regardless of changes in the **1/2/3** molar ratio. The static quenching observed for **2** is less pronounced than that of **1**, and this can be attributed to two possible reasons: 1) less efficient homo‐FRET for **2**; 2) larger average distance between **2**. Consequently, the calculated number of donors (**2**) quenched by a single acceptor (**3**) was determined to be only 6.9 (Figure S18a, Supporting Information). Additionally, exciton diffusion was assessed by plotting the 1/τ values of **2** versus the concentration of **3**. The second‐order exciton migration rate constant of **2**/**3** was determined to be as high as 1.32 × 10^15^ L mol^−1^ s^−1^ (Figure S18b, Supporting Information).

It is noteworthy that, in addition to the 2‐step sequential energy transfer process, direct energy transfer might occur from **1** to **3** (Figure 3d). To unravel the energy transfer efficiencies of each step, we calculated the *k*_ET_ rates for each energy transfer step. The FRET process of the two‐component systems, **1**/**2** and **1**/**3**, were initially studied, and the *k*_ET_ values for each process (*k*_ET, 1/2_, *k*_ET, 1/3_) were calculated (Figure S19 and Table S7, Supporting Information). As expected, both *k*_ET, 1/2_ and *k*_ET, 1/3_ increased with the rise in the **2**/**1** or **3**/**1** molar ratios. Furthermore, at the same donor/acceptor molar ratio, *k*_ET, 1/2_ and *k*_ET, 1/3_ values were found to be similar, consistent with their comparable spectral overlap (4.49 × 10^31^ m ^−1^ cm^−1^ nm^4^ vs 4.79 × 10^31^ m ^−1^ cm^−1^ nm^4^). Utilizing the information from the binary systems, we calculated the *k*_ET_ values for all possible energy transfer steps within the ternary system (Table S8, Supporting Information). Figure 3e summarizes the evolution of *k*_ET, 1/2_, *k*_ET, 1/3_, and *k*_ET, 2/3_ of the **1**/**2**/**3** system with the increase of **3** molar ratios. *k*_ET, 1/2_ remained almost constant ≈3.0 × 10^9^ s^−1^, while *k*_ET, 1/3_, and *k*_ET, 2/3_ increased rapidly upon the increase of **3** molar ratio. *k*_ET, 1/3_ was found to be larger than *k*_ET, 1/2_ when **1**/**2**/**3** molar ratio exceeded 100/10/8. *k*_ET_ values of the ternary system were greater than those in the binary systems of **1**/**2** and **1**/**3**. The energy transfer efficiencies of each step could then be determined. As depicted in Figure 3f, the molar ratio of **3** played a significant role in the contribution of *Φ*_ET, 1/2_ and *Φ*_ET, 1/3_ to the overall efficiency of **1**. *Φ*_ET, 1/2_ decreased from 90.5% to 36.3% as the **1**/**2**/**3** molar ratio varied from 100/10/0 to 100/10/10, while *Φ*_ET, 1/3_ exhibited a continuous increase from 0% to 60.7%. The overall energy transfer efficiency from **1** to **3** could also be determined (*Φ*_ET, overall_ = *Φ*_ET, 1/2_ × *Φ*_ET, 2/3_ + *Φ*_ET, 1/3_). Specifically, at a **1**/**2**/**3** molar ratio of 100/10/6, *Φ*_ET, 1/2_, and *Φ*_ET, 1/3_ values were calculated to be 52.1% and 43.8%, with a *Φ*_ET_, overall, of 91.9%. Therefore, despite that the direct energy from **1** to **3** could not be ignored in the ternary system, the high overall energy transfer efficiency ensures its outstanding performance as an artificial LHS.

### 2.4 Quaternary LHS with Three‐Step Sequential Energy Transfer

**Figure 4:**
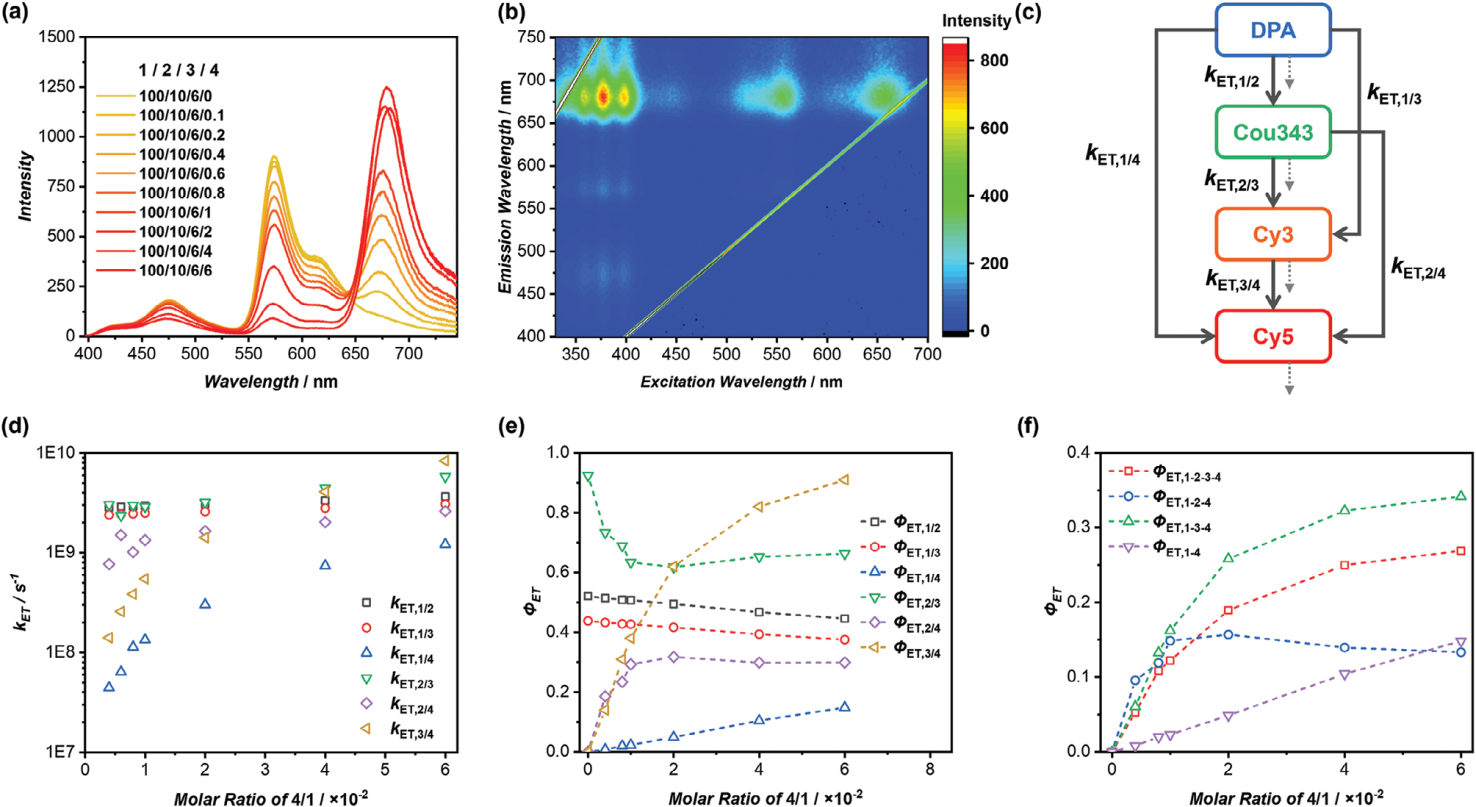
Energy transfer behaviors of the quaternary system of **1**/**2**/**3**/**4**. a) Fluorescence spectra of DPA‐CP‐PEG/Cou343‐CP/Cy3‐CP = 100/10/6 with different concentrations of Cy5‐CP (*λ*_ex_ = 377 nm); b) 2D excitation spectra of **1**/**2**/**3**/**4** (100/10/6/6); c) Energy transfer processes of the **1**/**2**/**3**/**4** quaternary system; d) Evolution of *k*_ET_ values of the **1**/**2**/**3**/**4** system with the increase of **4** molar ratios; e) Evolution of the *Φ*_ET_ values of the six individual energy transfer processes with the increase of **4** molar ratios; f) Evolution of the *Φ*_ET_ values of the four paths with the increase of **4** molar ratios.

We then explored the possibility of fabricating a 3‐step sequential energy transfer LHS by the incorporation of a third FRET acceptor, Cy5‐CP (**4**) (Figure S20, Supporting Information). To this end, a small amount of **4** was co‐assembled to the ternary system of **1**/**2**/**3** (100/10/6). Upon excitation at 377 nm, the emission of **3** centered at 573 nm attenuated, with the concomitant enhancement of a new emission band peaked at 677 nm ascribed to **4** (**Figure** **4**a; Figure S21, Supporting Information). The virtual Stokes shift is up to 300 nm for the quaternary system of **1**/**2**/**3**/**4**, highlighting the advantages of the multi‐step sequential energy transfer. The sequential energy transfer behaviors are further revealed by the 2D excitation spectrum of **1**/**2**/**3**/**4**. As shown in Figure 4b, when excited at wavelengths ranging from 350 to 675 nm, a large majority of the emission belongs to **4**, suggesting that **4** could effectively harvest the energy transferred from **1**, **2**, and **3**. The emission spectra were deconvoluted into 4 peaks of DPA, Cou343, Cy3, and Cy5 (Figures S22 and S23, Table S9, Supporting Information), allowing the precise evaluation of the performance of the system. As depicted in Figure S24 (Supporting Information), the *Φ*_ET_ value between **3**/**4** reached 90.5% at a **1/2/3/4** molar ratio of 100/10/6/6, along with a *Φ*_F_ value measured to be as high as 42.0%. The diffusion length of excitation energy from **3** to **4** is calculated to be 3.1 donor units, with a second‐order exciton migration rate constant up to 2.71 × 10^15^ L mol^−1^ s^−1^ (Figure S25, Supporting Information). Therefore, an efficient LHS with 3‐step sequential energy transfer was successfully constructed.

For the quaternary system of **1**/**2**/**3**/**4**, 6 individual energy transfer processes are possible: **1**→**2**, **1**→**3**, **1**→**4**, **2**→**3**, **2**→**4**, and **3**→**4** (Figure 4c). We successfully calculated the *k*_ET_ values for each process (Figure S26, Tables S10 and S11, Supporting Information). As summarized in Figure 4d, both *k*_ET, 1/2_ and *k*_ET, 1/3_ exhibited a 30% increase as the molar ratio of **4** increased from 0.2% to 6%, accompanied by a sharp increase of *k*_ET, 1/4_. The values for *k*_ET, 1/2_, *k*_ET, 1/3_, and *k*_ET, 1/4_ reached 3.66 × 10^9^, 3.08 × 10^9^, and 1.22 × 10^9^ s^−1^ at a **1**/**2**/**3**/**4** molar ratio of 100/10/6/6, implying that both **2**, **3**, and **4** could receive energy directly from **1** with **1**→**2** being the majority. Similarly, for *k*_ET_ values related to **2**, *k*_ET, 2/4_ boosted along the increase of **4**, while *k*_ET, 2/3_ only saw a slight increase. Finally, *k*_ET, 3/4_ increased markedly with the increase of **4** molar ratios. At a **1**/**2**/**3**/**4** molar ratio of 100/10/6/6, all 6 *k*_ET_ values exceeded 1 × 10^9^ s^−1^, guaranteeing the high energy transfer efficiency of the quaternary artificial light‐harvesting system.

For a more intuitive view of the quaternary system of **1**/**2**/**3**/**4**, the *Φ*_ET_ values of each step were determined (Table S12, Supporting Information). Figure 4e shows the evolution of the *Φ*_ET_ values of the 6 individual energy transfer processes. As expected, *Φ*_ET, 1/4_ increased with the increase of **4** molar ratio, accompanied by the decrease in both *Φ*_ET, 1/2_ and *Φ*_ET, 1/3_ values. However, given the fact that the spectral overlap between **1** and **4** was much smaller than either **1**/**2** or **1**/**3**, only 14.8% *Φ*_ET, 1/4_ was obtained at a **1**/**2**/**3**/**4** molar ratio of 100/10/6/6, while *Φ*_ET, 1/2_ and *Φ*_ET, 1/3_ values were 44.6%, and 37.5%, respectively. Interestingly, *Φ*_ET, 2/4_ increased from 0% to 31.8% as the **1**/**2**/**3**/**4** molar ratio varied from 100/10/6/0 to 100/10/6/2, followed by a slight decrease to 29.9% when the **1**/**2**/**3**/**4** molar ratio further increased to 100/10/6/6. Meanwhile, *Φ*_ET, 2/3_ exhibited the opposite trend as *Φ*_ET, 2/4_. The overall energy transfer from **1** to **4** could be cataloged into 4 paths: **1**→**2**→**3**→**4**, **1**→**2**→**4**, **1**→**3**→**4**, and **1**→**4**. As shown in Figure 4f, the *Φ*_ET_ values of the 4 paths were calculated. The *Φ*_ET_ value representing the 3‐step sequential energy transfer process constituted a part regardless of the **1**/**2**/**3**/**4** molar ratios. However, it is worth pointing out that while the direct energy transfer from **1** to **4** played a minor role, the two energy transfer processes (**1**→**2**→**4**, and **1**→**3**→**4**) involving two‐step sequential transfer are dominant within the quaternary system. At a **1**/**2**/**3**/**4** molar ratio of 100/10/6/6, *Φ*_ET, 1‐2‐3‐4_ was calculated to be 26.9%, while *Φ*_ET, 1‐2‐4_, *Φ*_ET, 1‐3‐4_, and *Φ*_ET, 1–4_ were 13.3%, 34.2%, and 14.8%, respectively. The overall *Φ*_ET_ reached as high as 89.2%. Therefore, through the co‐assembly of **1**/**2**/**3**/**4** utilizing the cyclic peptide‐based supramolecular scaffold, we have not only successfully constructed a highly efficient artificial LHS featuring a three‐step sequential energy transfer mechanism, but also meticulously investigated the associated energy transfer processes.

### 2.5 Quinary LHS with Four‐Step Sequential Energy Transfer

**Figure 5:**
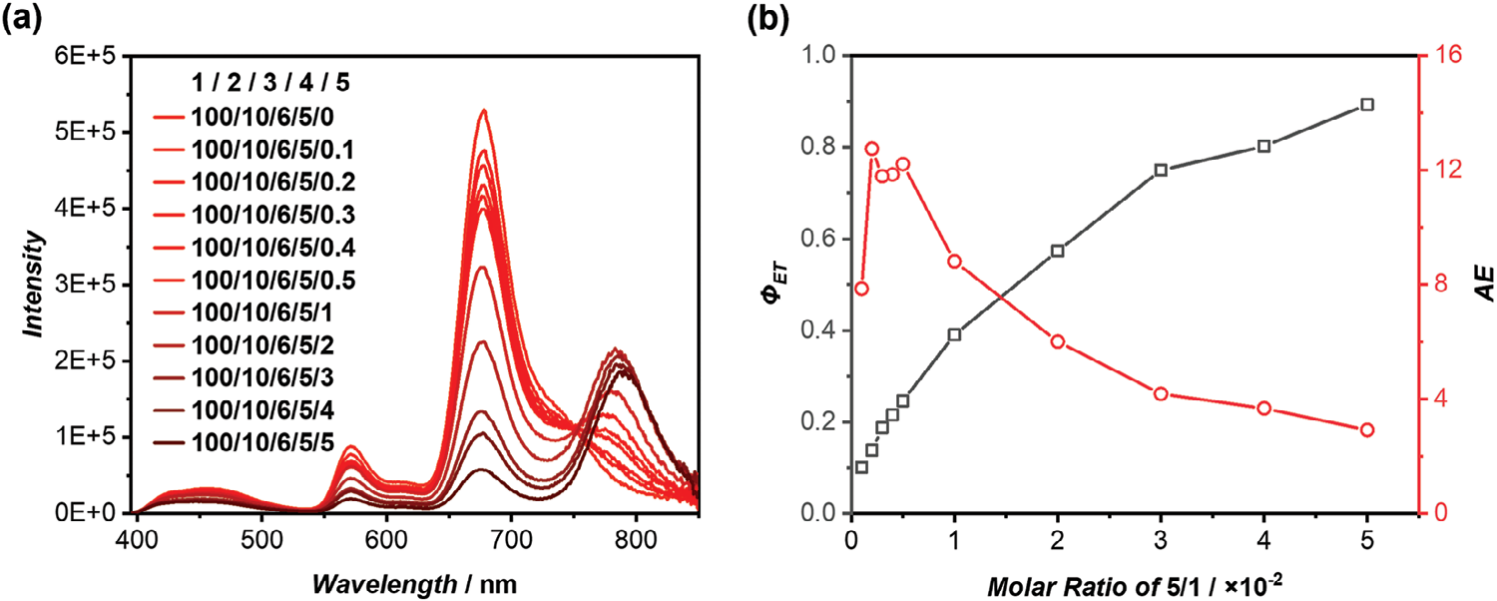
Energy transfer behaviors of the quinary system of **1**/**2**/**3**/**4**/**5**. a) Fluorescence spectra of DPA‐CP‐PEG/Cou343‐CP/Cy3‐CP/Cy5‐CP = 100/10/6/5 with different concentrations of Cy7‐CP (*λ*_ex_ = 377 nm); b) *Φ*_ET_ between **4**/**5** and *AE* values at different **5**/**1** molar ratios.

Encouraged by the impressive performance of the quaternary system involving a 3‐step sequential energy transfer process, we took on the challenge of constructing a 5‐component system that incorporates a 4‐step sequential energy transfer. To the best of our knowledge, there have been no reports of artificial LHSs featuring a four‐step sequential energy transfer. In pursuit of this goal, we introduced the fourth FRET acceptor, Cy7‐CP (**5**), into the existing quaternary system of **1**/**2**/**3**/**4** (Figure S27, Supporting Information). As illustrated in **Figure** **5**a, upon excitation at 377 nm, a new emission band peaked at 790 nm ascribed to Cy7 emerged. Simultaneously, the emission band of Cy5 gradually decreased with an increase in the molar ratio of **5**. The calculated virtual Stokes shift was 413 nm. At a **1**/**2**/**3**/**4**/**5** molar ratio of 100/10/6/5/5, the *Φ*_ET_ value between **4**/**5** reached an impressive 89.3% with a *Φ*_F_ value of 17.6% (Figure 5b). This highlights the remarkable energy transfer efficiency achieved in the cyclic peptide‐based artificial LHSs. Due to the complexity of the quinary system, the analysis of the related energy transfer pathways was not conducted, yet the calculation methodology remains identical to that of the quaternary system.

## 3 Conclusion

We have reported the construction of artificial LHSs incorporating a multi‐step sequential energy transfer mechanism. The densely arranged fluorophores facilitated both direct hetero‐energy transfer between the donor/acceptor pairs and homo‐energy transfer within donors before transferring energy to the acceptor, yielding a series of artificial LHSs distinguished by their exceptional energy transfer efficiency, large diffusion length of excitation energy, and elevated exciton migration rate. More importantly, the intricate energy transfer pathways within these LHSs were elucidated. Therefore, the cyclic peptide‐based supramolecular scaffold not only yields a suite of high‐performance artificial LHSs, but also offers critical insights for the design of advanced artificial LHSs.

## Conflict of Interest

The authors declare no conflict of interest.

## Supporting information

Supporting Information

## Data Availability

The data that support the findings of this study are available from the corresponding author upon reasonable request.
