# Supplementary material for: Comprehensive Study of Artificial Light‐Harvesting Systems with a Multi‐Step Sequential Energy Transfer Mechanism
Source: Adv Sci (Weinh). 2024 Jun 14;11(31):2404269. doi: 10.1002/advs.202404269 (PMC11336932; doi:10.1002/advs.202404269)
Supplement: Supplementary file 1 — Supporting Information [file ADVS-11-2404269-s001.docx]

**Supporting Information**

**Comprehensive Study of Artificial Light-Harvesting Systems with a Multi-Step Sequential Energy Transfer Mechanism**

Yong Wu,^[a]^ Yuqian Wang,^[a]^ Xu Yu,^[b]^ and Qiao Song^*[a,c]^

[a] Y. Wu, Y. Wang, Dr. Q. Song

Shenzhen Grubbs Institute, Southern University of Science and Technology

Shenzhen 518055 (China)

E-mail: songq@sustech.edu.cn

[b] Prof. X. Yu

Institute of Innovation Materials and Energy, College of Chemistry and Chemical Engineering, Yangzhou University

Yangzhou 225002 (China)

[c] Dr. Q. Song

Guangming Advanced Research Institute, Southern University of Science and Technology

Shenzhen 518055 (China)

**S1. Materials and Characterization**

**Materials**

Linear peptide (H_2_N-L-Lys(Dde)-D-Leu-L-Trp(Boc)-D-Leu-L-Lys(Boc)-D-Leu-L-Trp(Boc)-D-Leu-COOH) was purchased from KS-V Peptide. Acetic acid-terminated PEG derivative (CH_3_O-PEG-COOH, *M*_n_=5000 g mol^-1^) was purchased from JenKem. Cou343-CP, Cy3-CP, Cy5-CP, and Cy7-CP were obtained from our previous work.^[12a-b]^ 4-(10-Phenylanthracen-9-yl)benzoic acid (DPA-COOH), *O*-(7-azabenzotriazol-1-yl)-*N*,*N*,*N*',*N*'-tetramethyl uronium hexafluorophosphate (HATU), *N*-methylmorpholine (NMM), 4-(4,6-Dimethoxy-1,3,5-triazin-2-yl)-4-methylmorpholin-4-ium tetrafluoroborate (DMTMM·BF_4_) and other chemicals were purchased from several suppliers, including Bidepharm, J&K, and Sigma-Aldrich. Solvents were purchased from several local suppliers, including General-Reagent, and J&K.

**Characterization**

**Nuclear Magnetic Resonance Spectroscopy (NMR)**: ^1^H NMR spectra were measured using a Bruker Avance III HD 400 MHz NMR spectrometer with dimethyl sulfoxide-*d*_6_ (DMSO-*d*_6_) as the solvent. The residual solvent peaks were used as internal references.

**Liquid Chromatography-Mass Spectrometry (LC-MS)**: LC-MS analysis was conducted using a WATERS H-Class/QDa Mass Spectrometer coupled with a WATERS ACQUITY UPLC to characterize the chemicals in positive ion mode. Water and acetonitrile were used as mobile phase A and B, respectively. All solvents contained 0.1 % (v/v) formic acid. Samples were dissolved in mobile phase B with a final concentration of 0.1 mg mL^-1^ and the injection volume was 2 µL.

**Matrix-Assisted Laser Desorption/Ionization-Time of Flight Mass Spectrometry (MALDI-TOF MS):** MALDI-TOF MS measurements were conducted using Bruker Autoflex Speed LRF, equipped with a 355 nm Nd:YAG laser. DCTB was used as a matrix (20 mg mL^-1^ in acetonitrile).

**Ultraviolet–Visible (UV–Vis) Absorption Spectroscopy**: UV–Vis absorption spectra were measured using a SHIMADZU UV-2600i UV–vis spectrometer. Unless otherwise stated, the path length of the cuvette was 10 mm.

**Fluorescence Emission Spectroscopy**: Fluorescence emission spectra were measured using either an Edinburgh Instruments FLS1000 photoluminescence spectrometer or a HITACHI F-4700 fluorescence spectrometer.

**Time-resolved Fluorescence Spectroscopy**: Fluorescence lifetime measurements were performed using an Edinburgh Instruments FLS1000 photoluminescence spectrometer, equipped with several Pulsed Lasers - EPL Series. The measurements were conducted at room temperature.

**Fluorescence Quantum Yield (*Φ*_F_)**: These measurements were performed based on the protocol proposed by Würth et al.^[14]^ for determining the fluorescence quantum yields of transparent samples, with 9,10-diphenylanthracene used as a reference sample.^[15]^

**Small Angle Neutron Scattering (SANS)**: SANS was carried out at the SANS instrument in China Spallation Neutron Source (CSNS). The sample to detector distance was set to 4 m, with a wavelength band from 1 Å to 9.8 Å, yielding a *q*-range of 0.005 Å^-1^ – 0.9^-1^ Å^-1^. Samples with deuterated solvent were loaded in the Hellma quartz cells with 2 mm light path and measured at room temperature. The scattering profiles of the samples have been calibrated to absolute scaling with sample transmission and a secondary standard sample (Bates-poly) provided by the beamline. The corresponding background contributions including solvent, empty cell as well as empty beam data were also collected and properly subtracted before data analysis.

**S2. Synthesis and Self-assembly**

**a. Synthesis of Cyclic Peptide**


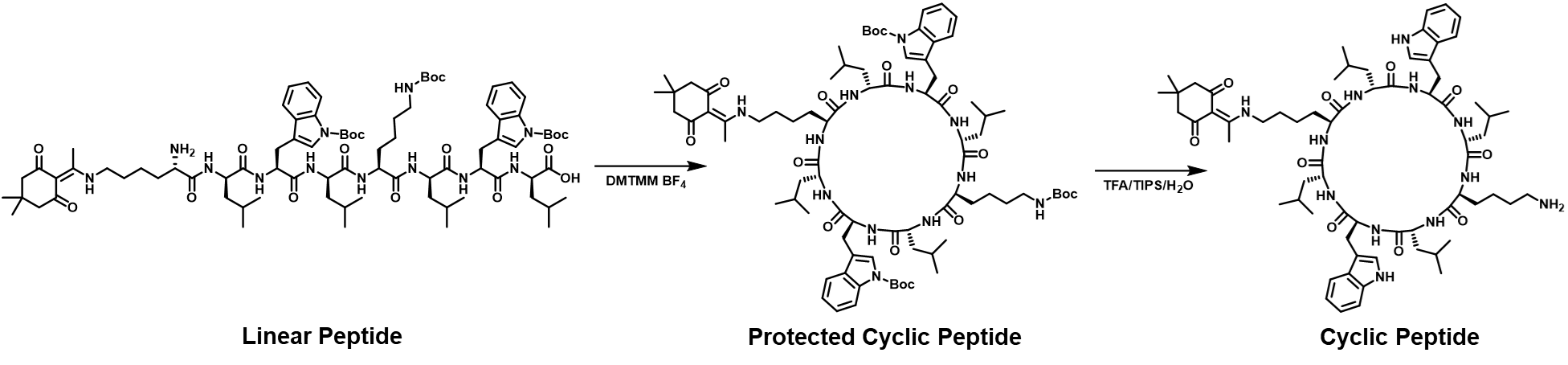


*Cyclization*: Linear peptide was cyclized by stirring at room temperature for 3 days in the presence of 1.2 equivalents of DMTMM·BF_4_ in 100 mL DMF under the protection of N_2_. The solution was then concentrated to 10 mL under reduced pressure and then precipitated with cold methanol/water=1/1 to obtain a white powder as protected cyclic peptide. Yield: 64%.

*Deprotection*: Removal of the -Boc protecting groups was achieved by adding a mixture of trifluoroacetic acid (TFA, 900 μL), triisopropylsilane (TIPS, 50 μL) and water (50 μL) to the protected cyclic peptide and stirring for 3 hours. The resulting solution was then precipitated in ice cold diethyl ether and washed twice to give an off-white powder as the targeted **Cyclic Peptide**. Yield: 91%. (m/z=1245.6, [C_68_H_101_N_12_O_10_]^+^: 1245.8).

**b. Synthesis of DPA-CP-PEG**


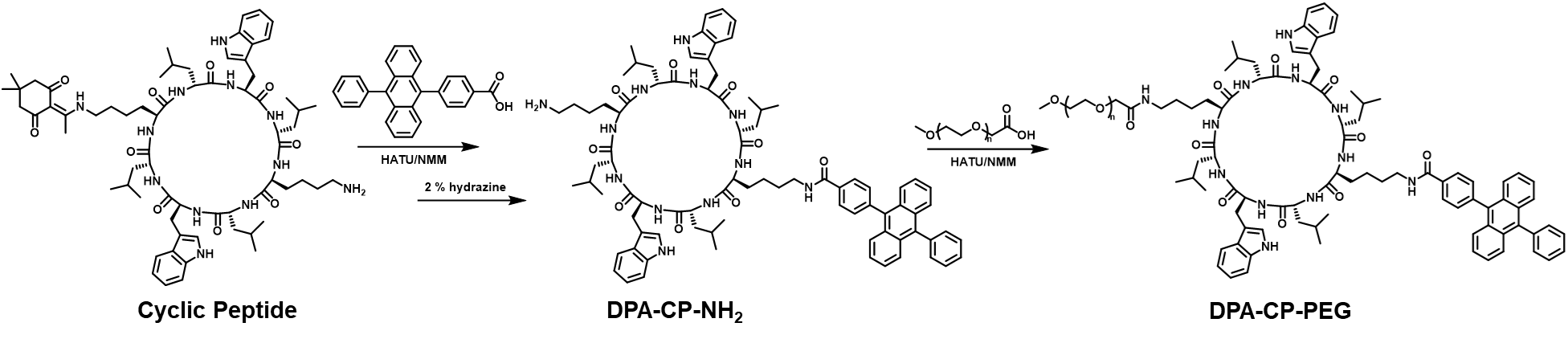


*Synthesis of* **DPA-CP-NH_2_**: **Cyclic Peptide** (20.0 mg, 16.06 μmol) and DPA-COOH (9.0 mg, 24.08 μmol) were dissolved in 1.0 mL DMF, with the addition of HATU (9.2 mg, 24.08 μmol) and NMM (4.9 mg, 48.16 μmol). The reaction was left overnight. The DMF solution was then precipitated in a mixed solvent of diethyl ether: THF = 1:3 and washed twice to obtain DPA-CP-NH(Dde) (yield: 17.5 mg, 68%). The vacuum-dried solid (15.0 mg, 9.36 μmol) was redissolved in 1 mL DMF containing 2% hydrazine and stirred for 1 h. The reaction mixture was precipitated in diethyl ether and washed twice. The obtained precipitate was dried under vacuum to obtain **DPA-CP-NH_2_** (yield: 11.4 mg, 85%).


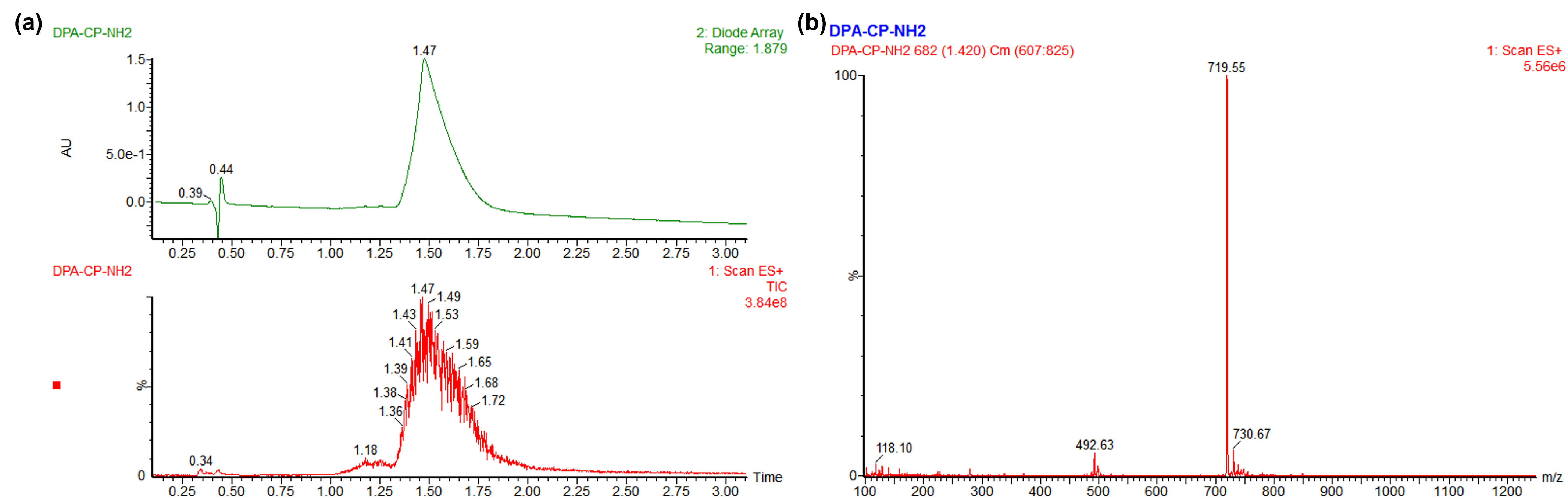


Figure S1 LC-MS analysis of **DPA-CP-NH_2_** (a) LC profile using UV detector (top) and MS detector (bottom); (b) mass spectrum of the LC peak (m/z=719.6, [C_85_H_106_N_12_O_9_]^2+^: 719.4).

*Synthesis of* **DPA-CP-PEG**: **DPA-CP-NH_2_** (10.0 mg, 6.95 μmol) and CH_3_O-PEG-COOH (45.2 mg, 9.04 μmol) were dissolved in 1.0 mL DMF, with the addition of HATU (4.0 mg, 10.43 μmol) and NMM (2.1 mg, 20.86 μmol). The reaction was left overnight. The DMF solution was then precipitated in a mixed solvent of diethyl ether: DCM = 5:1 and washed twice to obtain **DPA-CP-PEG** (yield: 29.0 mg, 65%).


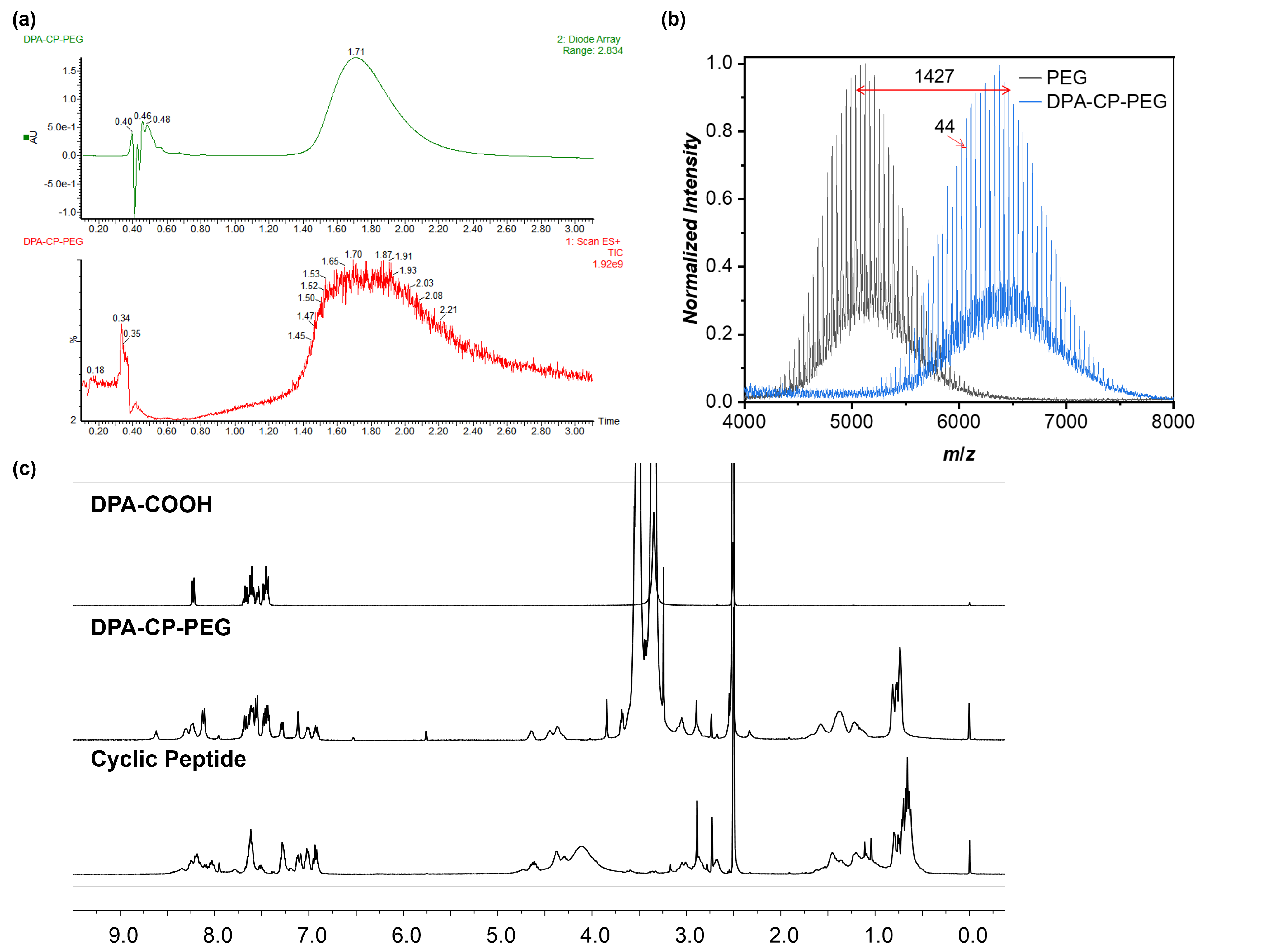


Figure S2 Characterization of **DPA-CP-PEG** (a) LC-MS of **DPA-CP-PEG**; (b) MALDI-TOF MS of **DPA-CP-PEG** and CH_3_O-PEG-COOH; (c) ^1^H NMR spectra of **DPA-CP-PEG**, DPA-COOH, and cyclic peptide (400 MHz, DMSO-*d*_6_).

**c. Synthesis of Fluorophore-CP Conjugates**

Cou343-CP, Cy3-CP, Cy5-CP, and Cy7-CP were obtained from our previous work.^[12a-b]^


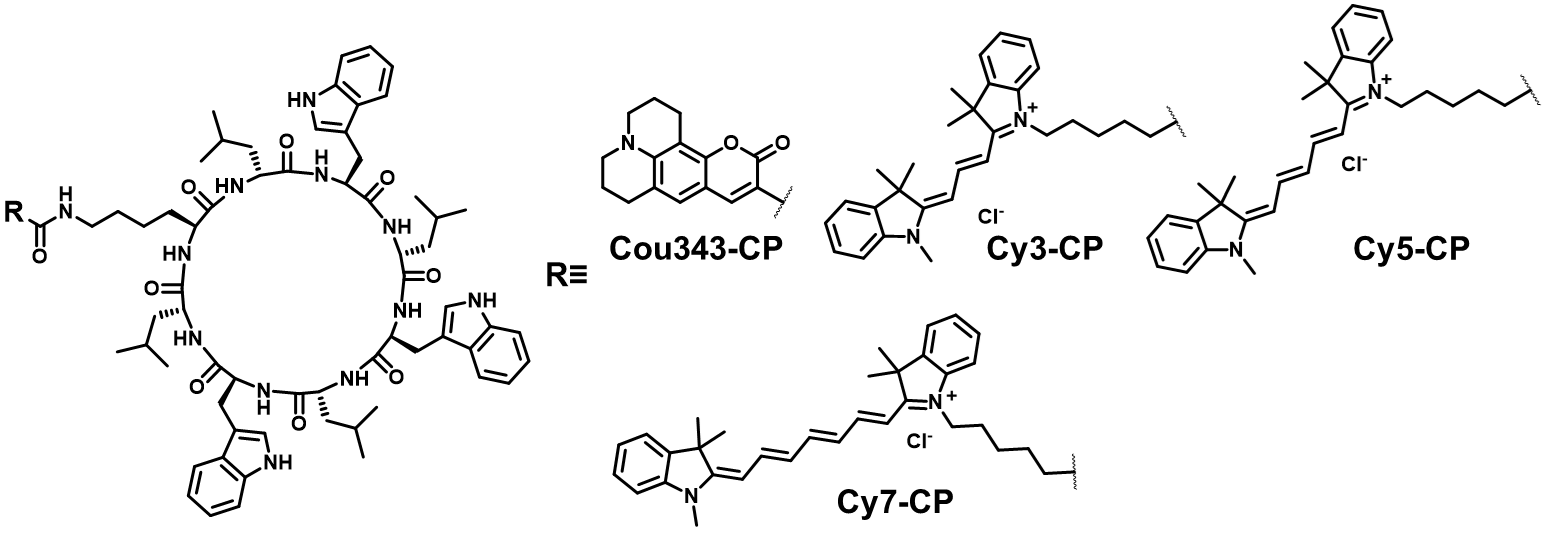


**d. Sample preparation**

The self-assembly of DPA-CP-PEG was realized by firstly dissolving it in a small amount of DMSO, followed by the addition of DI water, resulting clear solutions with H_2_O/DMSO ratio of 95/5. In the case of SANS, deuterated solvents were used (i.e. DMSO-*d*_6_ instead of DMSO, D_2_O instead of H_2_O).

For the co-assembly of DPA-CP-PEG with the acceptor conjugates (preparation of ALHSs), conjugates were premixed at certain molar ratios in DMSO before adding DI water to obtain solutions with desired concentrations while keeping H_2_O/DMSO ratio as 95/5. The solutions were kept in the dark at room temperature before measurements. Unless otherwise stated, the concentration of DPA-CP-PEG in the ALHSs was 10 μM.

**e. Self-assembling behavior**

SASfit software was used to fit the SANS data, using a cylindrical micelle model. This model describes a cylindrical micelle core, which in this case the core corresponds to the cyclic peptide, and a corona modelled as Gaussian chains of a given volume. Here, the corona is assumed to consist of PEG with the volume calculated based on the polymer molecular weight. SLD values were calculated using based on the atomic composition of the conjugate and solvent and molecular volumes calculated from mass densities (for solvents and ethylene glycol) or literature values for amino acid residues within the cyclic peptide. The fitting procedure was performed to minimize the reduced *χ*^2^, which is normalized by the number of data points and the number of fitting parameters.

Table S1 Fitting parameters using a cylindrical micelle model implemented with SASfit software.

| **Model** | **Parameters** | **DPA-CP-PEG** |
| --- | --- | --- |
| CYL+Chains(RW) | Core radius* / Å | 5 |
|  | Grafting density / Å^-2^ | 0.026±0.001 |
|  | Brush volume* / Å^3^ | 7380 |
|  | SLD_Core_* / ×10^-6^ Å^-2^ | 2.03 |
|  | SLD_Brush_* / ×10^-6^ Å^-2^ | 0.64 |
|  | SLD_Solvent_* / ×10^-6^ Å^-2^ | 6.39 |
|  | *R*_g,Brush_ / Å | 36.5±0.2 |
|  | Core penetration* | 1 |
|  | Length / Å | 141.8±4.0 |
|  | Scale | 0.25±0.04 |
| Extended Guinier law | *I*_0_ | 0.014 |
|  | *α** | 2 |
|  | *R*_g_ | 12.9±0.9 |
|  | *N* | 0.0014 |
| *χ*^2^ | | 4.77 |

Parameters marked with * were held constant throughout the fitting procedure, mean ± SD.

**S3. Binary LHS with One-Step Energy Transfer**


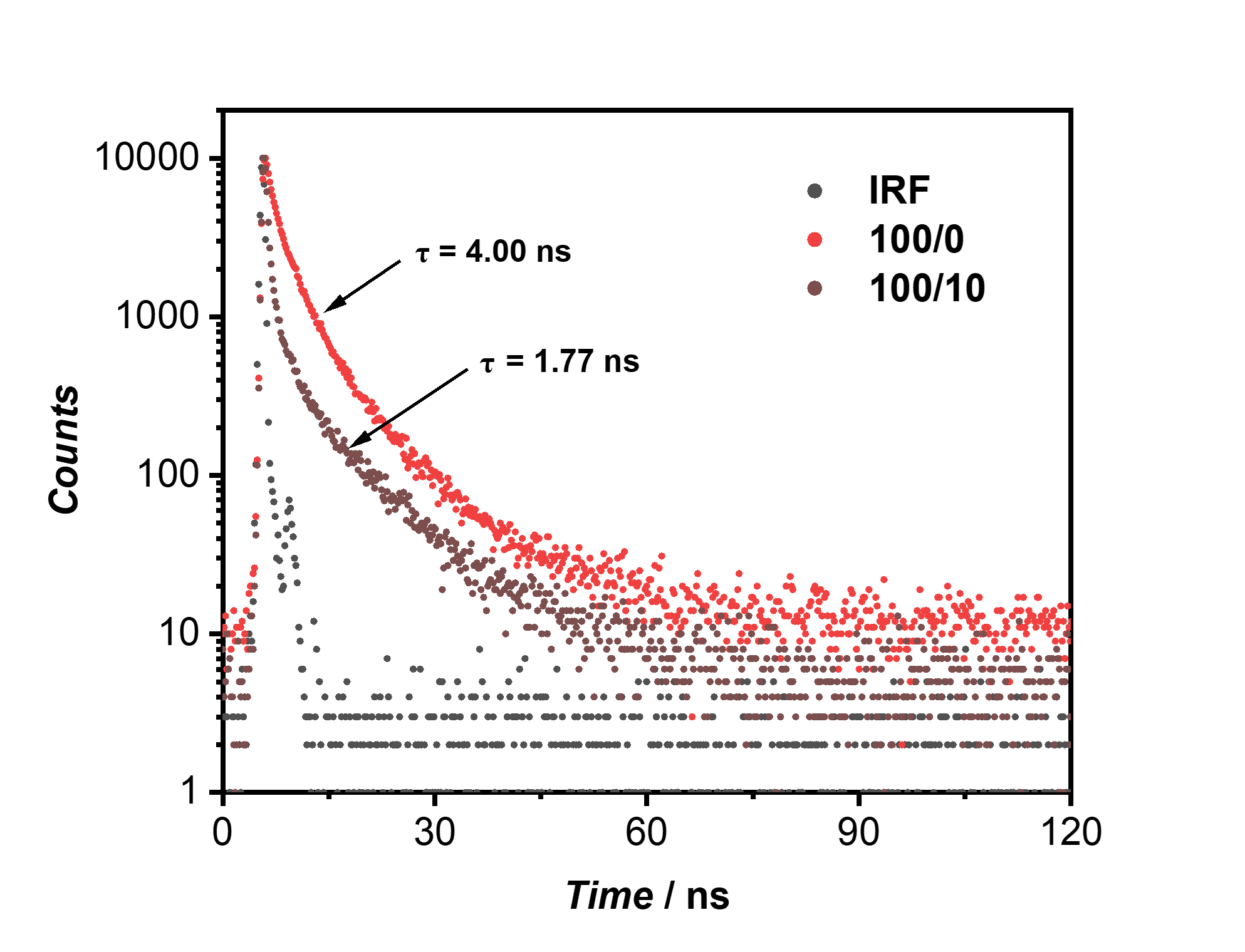


Figure S3 Time-resolved fluorescence spectra of **1** and **1**/**2** (100/10) monitored
at 435 nm ([**1**]=10 μM).

*Deconvolution of the emission spectra of* ***1****/****2***: Mathematical deconvolution was employed to separate the emission spectra. Assuming that the shape of the DPA emission spectrum remained constant, it was rescaled according to the emission spectra of **1**/**2**. The emission spectrum of Cou343 was then derived by excluding the rescaled DPA emission from the whole spectrum (Figure S4). This process enabled the isolation of the emission spectra of DPA and Cou343 within the **1**/**2** FRET system (Figure S5). Integrating the corresponding spectra provided the intensities of DPA (*I*_DPA_), Cou343 (*I*_Cou343_), and the overall (*I*_total_) emission (Table S2).


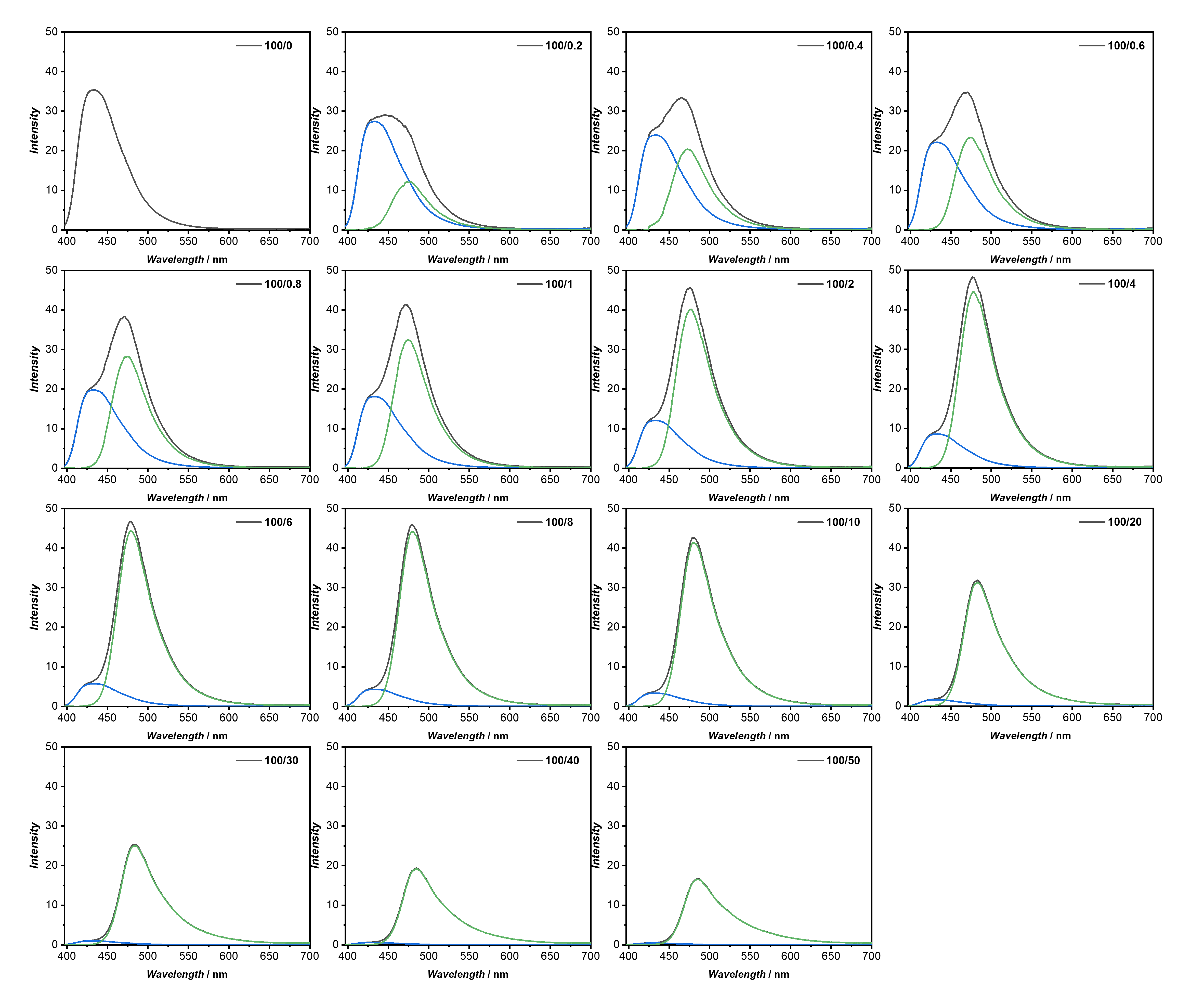


Figure S4 Deconvolution of emission spectra of **1**/**2** (grey) into emission bands of DPA (blue), and Cou343 (green).


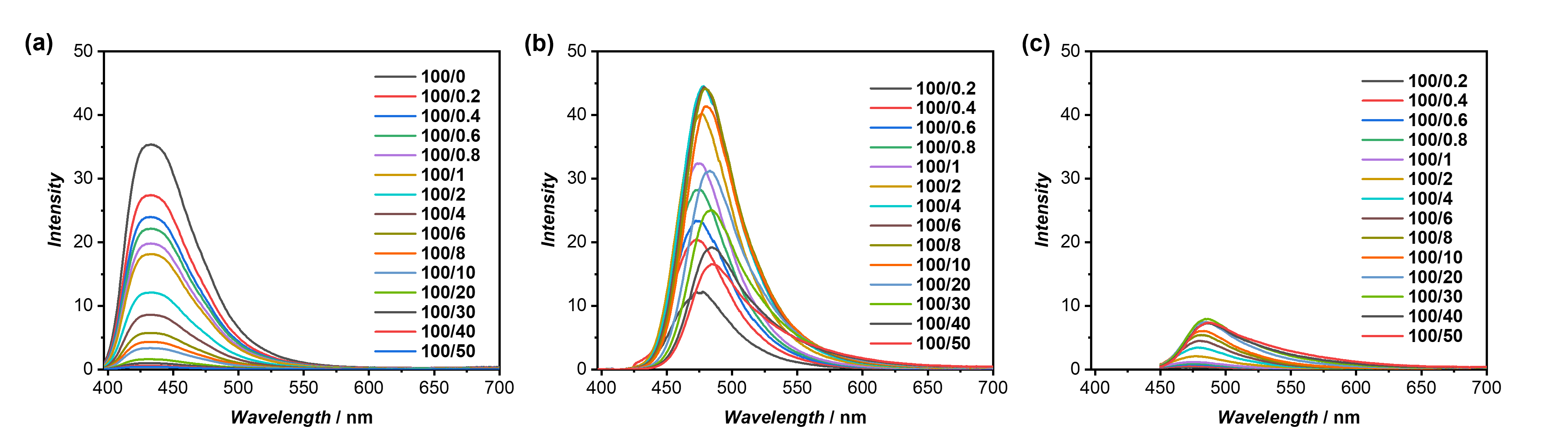


Figure S5 Deconvoluted emission spectra of DPA (a) and Cou343 (b); emission spectra of Cou343 when excited at 430 nm (c).


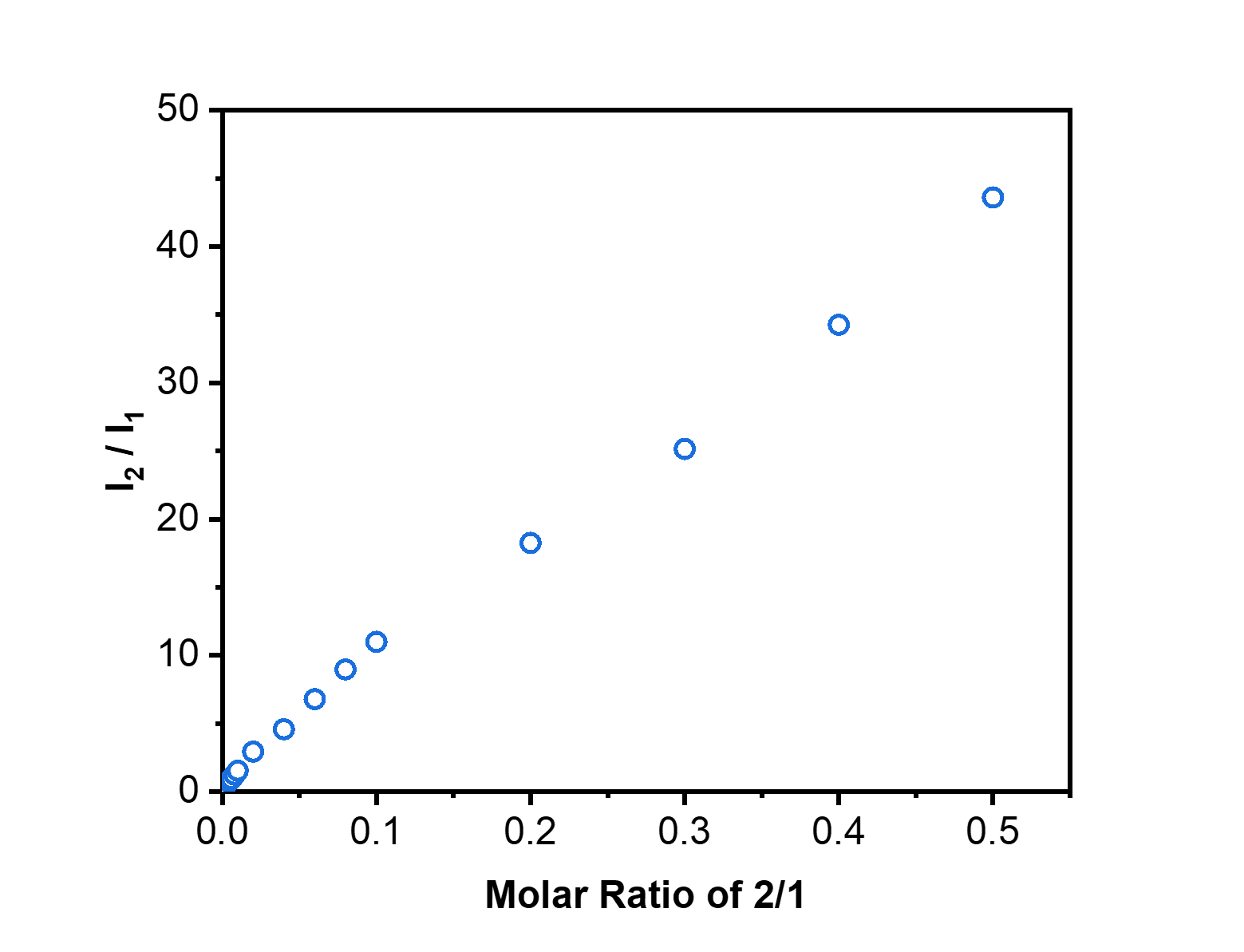


Figure S6 Ratiometric plot of ***I*_2_**/***I*_1_**.


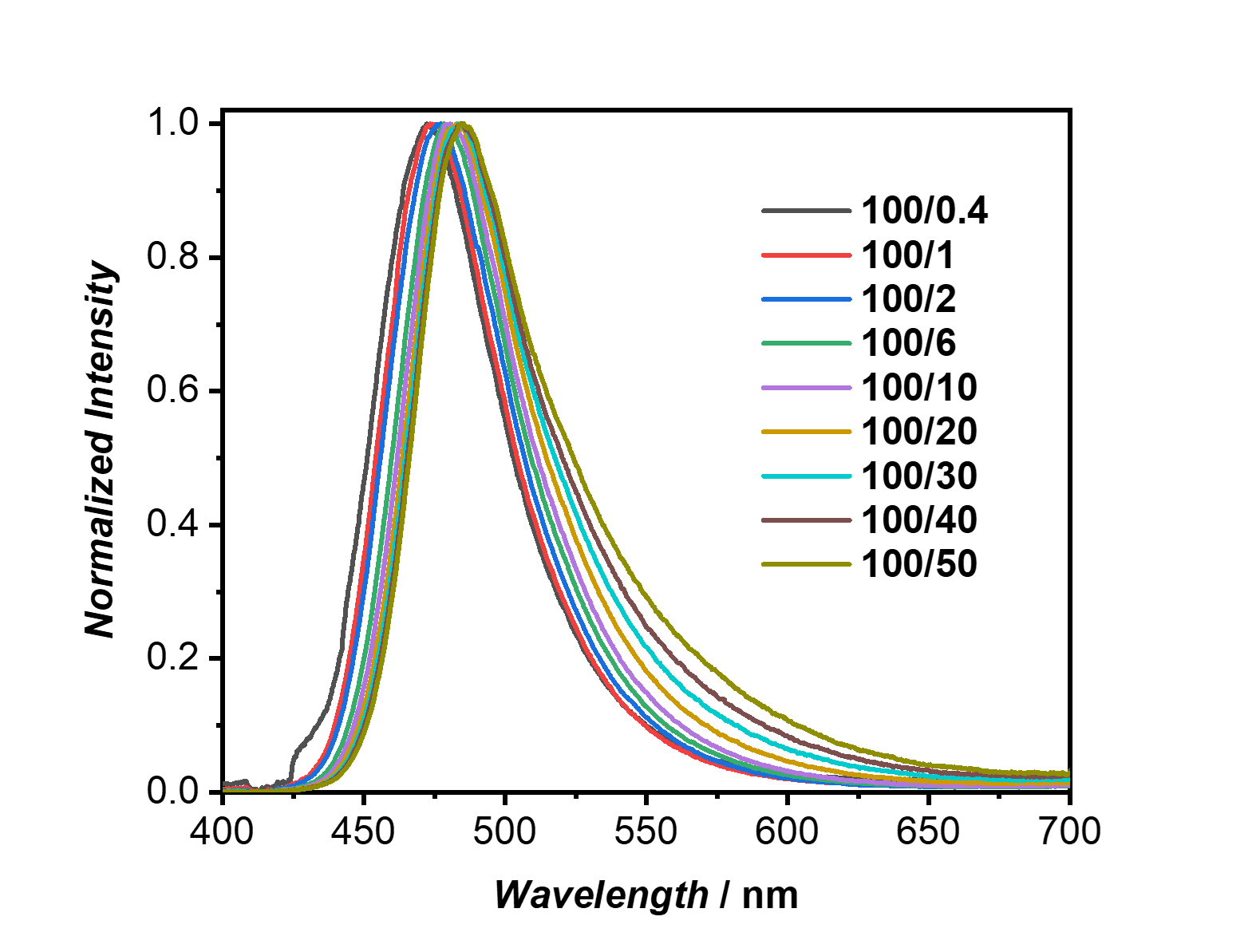


Figure S7 Normalized emission spectra of Cou343.

*Calculation of energy transfer efficiency (Φ_ET_) and antenna effect (AE)*: The *Φ*_ET_ and *AE* values were calculated using the equations shown below:

*Φ*_ET_ = 1-*I*_DPA(100/0)_ / *I*_DPA_

*AE* = *I*_Cou343_ / *I*_Cou343,direct_

Where *I*_Cou343,direct_ is the integral of the emission spectrum of Cou343 when excited at 430 nm (Figure S5c).

*Calculation of static and dynamic quenching*: The total fluorescence quenching efficiency (*η*_tot_) equals to the sum of dynamic quenching efficiency (*η*_dyn_) and static quenching efficiency (*η*_stat_). The values for static quenching and dynamic quenching were derived using the equations presented below:

*η*_dyn_ = 1- *τ*/*τ*_0_

*η*_tot_ = *Φ*_ET_ = 1-*I*_DPA(100/0)_ / *I*_DPA_

*η*_stat_ = *η*_tot_ - *η*_dyn_

Table S2 The intensities of DPA (*I*_DPA_), Cou343 (*I*_Cou343_), overall (*I*_total_) emission and calculated *Φ*_ET_ and *AE* values.

|  | *I*_total_ | *I*_DPA_ | *I*_Cou343_ | *Φ*_ET_ | *I*_Cou343,direct_ | *AE* |
| --- | --- | --- | --- | --- | --- | --- |
| 100/0 | 2393 | 2393 | - | - | - | - |
| 100/0.2 | 2586 | 1854 | 731 | 0.225 | 46 | 25.1 |
| 100/0.4 | 2848 | 1622 | 1226 | 0.322 | 56 | 29.1 |
| 100/0.6 | 2901 | 1497 | 1404 | 0.374 | 70 | 25.7 |
| 100/0.8 | 3013 | 1340 | 1673 | 0.440 | 79 | 26.1 |
| 100/1 | 3128 | 1227 | 1900 | 0.487 | 93 | 24.6 |
| 100/2 | 3223 | 818 | 2405 | 0.658 | 145 | 18.3 |
| 100/4 | 3242 | 581 | 2661 | 0.757 | 224 | 12.6 |
| 100/6 | 3023 | 388 | 2635 | 0.838 | 284 | 9.6 |
| 100/8 | 2923 | 293 | 2630 | 0.878 | 342 | 7.9 |
| 100/10 | 2723 | 227 | 2496 | 0.905 | 381 | 6.7 |
| 100/20 | 2079 | 108 | 1971 | 0.955 | 495 | 4.1 |
| 100/30 | 1727 | 66 | 1660 | 0.972 | 562 | 3.0 |
| 100/40 | 1375 | 39 | 1336 | 0.984 | 557 | 2.5 |
| 100/50 | 1249 | 28 | 1221 | 0.988 | 613 | 2.1 |


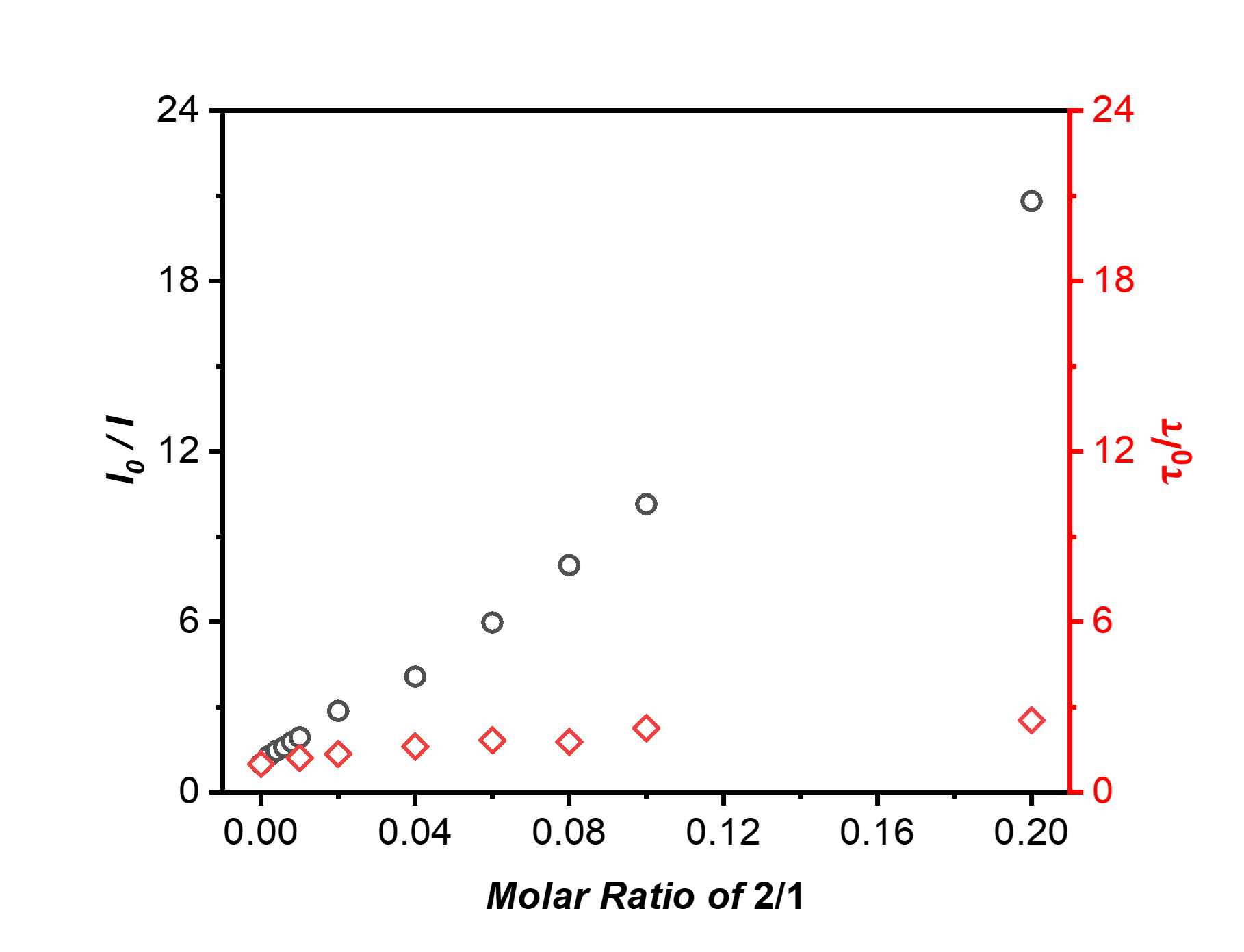


Figure S8 Stern-Volmer plots of the steady-state fluorescence intensity of **1** and the average fluorescence lifetime of **1**.

Table S3 Dynamic and static quenching of **1**/**2** upon varying the molar ratios of **1**/**2**.

| **1**/**2** | *Φ*_ET_ | *τ*_avg_ / ns | *η*_tot_ | *η*_dyn_ | *η*_stat_ |
| --- | --- | --- | --- | --- | --- |
| 0 | - | 4.00 | - | - | - |
| 100/1 | 0.487 | 3.30 | 48.7% | 17.5% | 31.2% |
| 100/2 | 0.658 | 2.95 | 65.8% | 26.1% | 39.8% |
| 100/4 | 0.757 | 2.47 | 75.7% | 38.1% | 37.7% |
| 100/6 | 0.838 | 2.18 | 83.8% | 45.5% | 38.3% |
| 100/8 | 0.878 | 2.24 | 87.8% | 43.8% | 43.9% |
| 100/10 | 0.905 | 1.77 | 90.5% | 55.8% | 34.7% |
| 100/20 | 0.955 | 1.57 | 95.5% | 60.7% | 34.8% |
| 100/30 | 0.972 | 1.43 | 97.2% | 64.1% | 33.1% |
| 100/40 | 0.984 | 1.35 | 98.4% | 66.3% | 32.1% |
| 100/50 | 0.988 | 1.12 | 98.8% | 71.9% | 26.9% |

*Determination of the number of donors (n) that can be quenched by a single acceptor*: Considering the contributions of both dynamic and static quenching in the donor quenching process, a model integrating both mechanisms was employed to compute the *n* value. The model assumes a 1:1 binding isotherm, implying that one FRET donor binds with one acceptor. The expression for this model is represented as follows:

(Donor)_n_ + Acceptor = (Donor)_n_-Acceptor

Expanding on this groundwork, a donor quenching model was applied, delineated by the equation below:

$$I_{F}=I_{0}-\frac{I_{0}}{2c_{0}}\times\left\{ \left( c_{0}+c_{A}+\frac{1}{K_{a}} \right)-\left( \left( c_{0}+c_{A}+\frac{1}{K_{a}} \right)^{2}-4c_{0}c_{A} \right)^{\frac{1}{2}} \right\}$$

For the equation, *I*_F_ is the emission intensity of **1** within the **1**/**2** system. *I*_0_ is the emission intensity of **1** in the absence of **2**. *c*_0_ denotes the concentration of (Donor)_n_, while *c*_A_ is the concentration of **2**. *K_a_* is the association constant on the basis of the 1:1 binding model.

By applying non-linear fitting of the emission intensities of **1** (*I*_F_) against the concentration of **2** (*c*_A_), the value of *c*_0_ was determined to be 3.97×10^-9^ M, which gave *n* = *c*(**1**) /*c*_0_ = 252.

*Note*: a 10-fold dilution was employed for a precise determination of the *c*_0_ value.

*Calculation of the second-order exciton migration rate constant*: Recognizing the pivotal role of the exciton migration rate in analyzing exciton migration behaviors, we evaluated this parameter for the energy transfer systems, considering both radiative relaxation and exciton trapping. The intricate processes involved can be elucidated as follows:

D^*^ + A → A^*^ + D (Reaction rate: *k*)

D^*^ → D (Reaction rate: *k*_0_)

Where D and D^*^ represent the ground state and the excited state of donors, respectively. A and A^*^ represent the ground state and the excited state of acceptor units, respectively. Accordingly, the quenching rate of exciton can be expressed as follows:

$$\frac{d[D^{*}]}{dt}=-k\left[ D^{*} \right]\left[ A \right]-k_{0}\left[ D^{*} \right]$$

In fluorescence lifetime decay measurements, the excitation power of the light source is consistently kept at a low level. Therefore, it is assumed that the concentration of excited donor is exceedingly low compared to that of the ground state acceptor ([A]>>[D^*^]). Thus, the concentration of excitons can be described using the following equation:

$${[D}^{*}]=e^{\frac{-t}{\tau}}$$

The decay of excitons adheres a mono-exponential quenching rate. By plotting the reciprocal of fluorescent lifetimes (1/τ) of **1** against the concentration of **2**, the second-order rate constant for the exciton migration process is equivalent to the slope of the linearly fitted line.

**S4. Ternary LHS with Two-Step Sequential Energy Transfer**


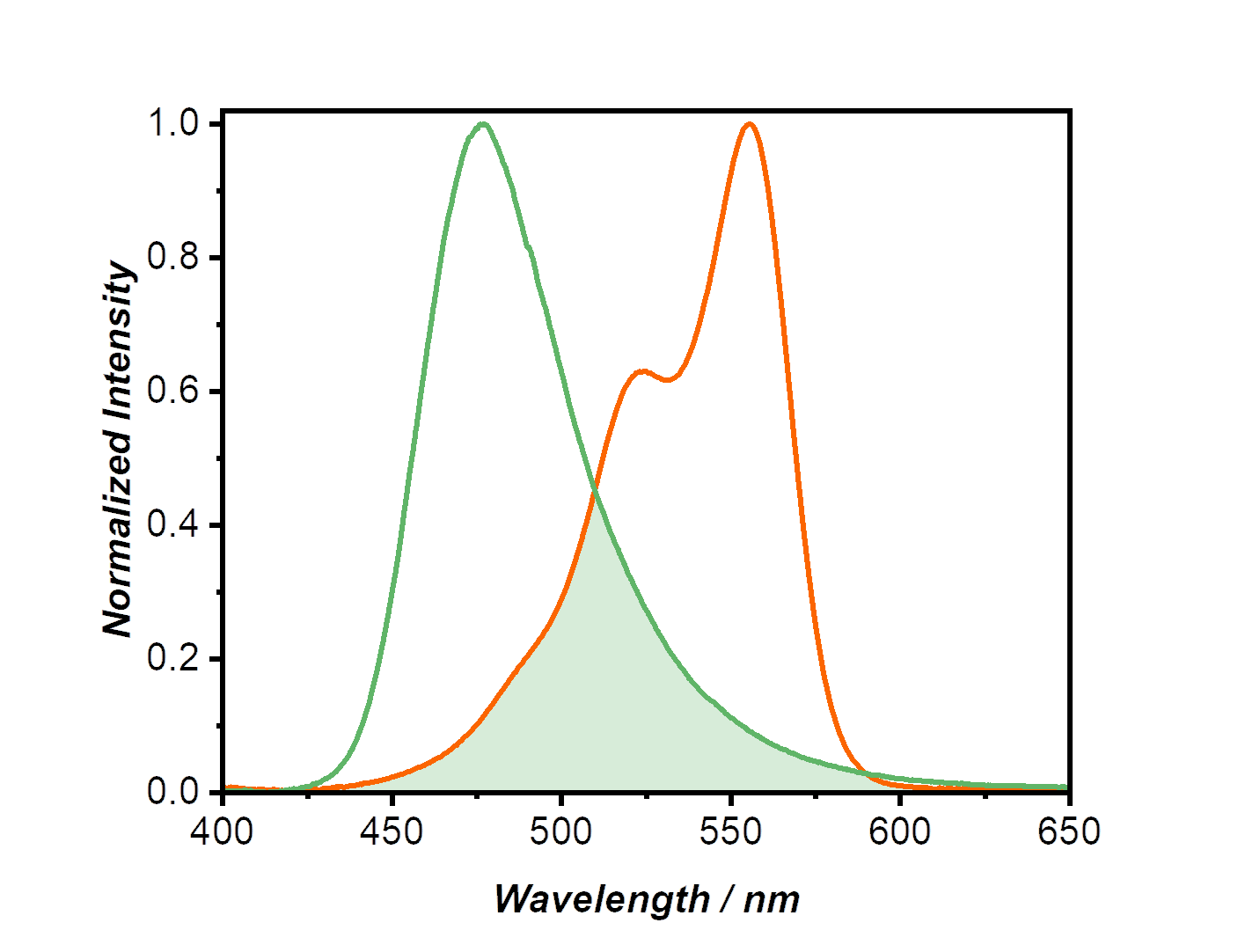


Figure S9 Spectral overlap of the normalized fluorescence spectrum of **2** (green)
and absorption spectrum of **3** (orange).


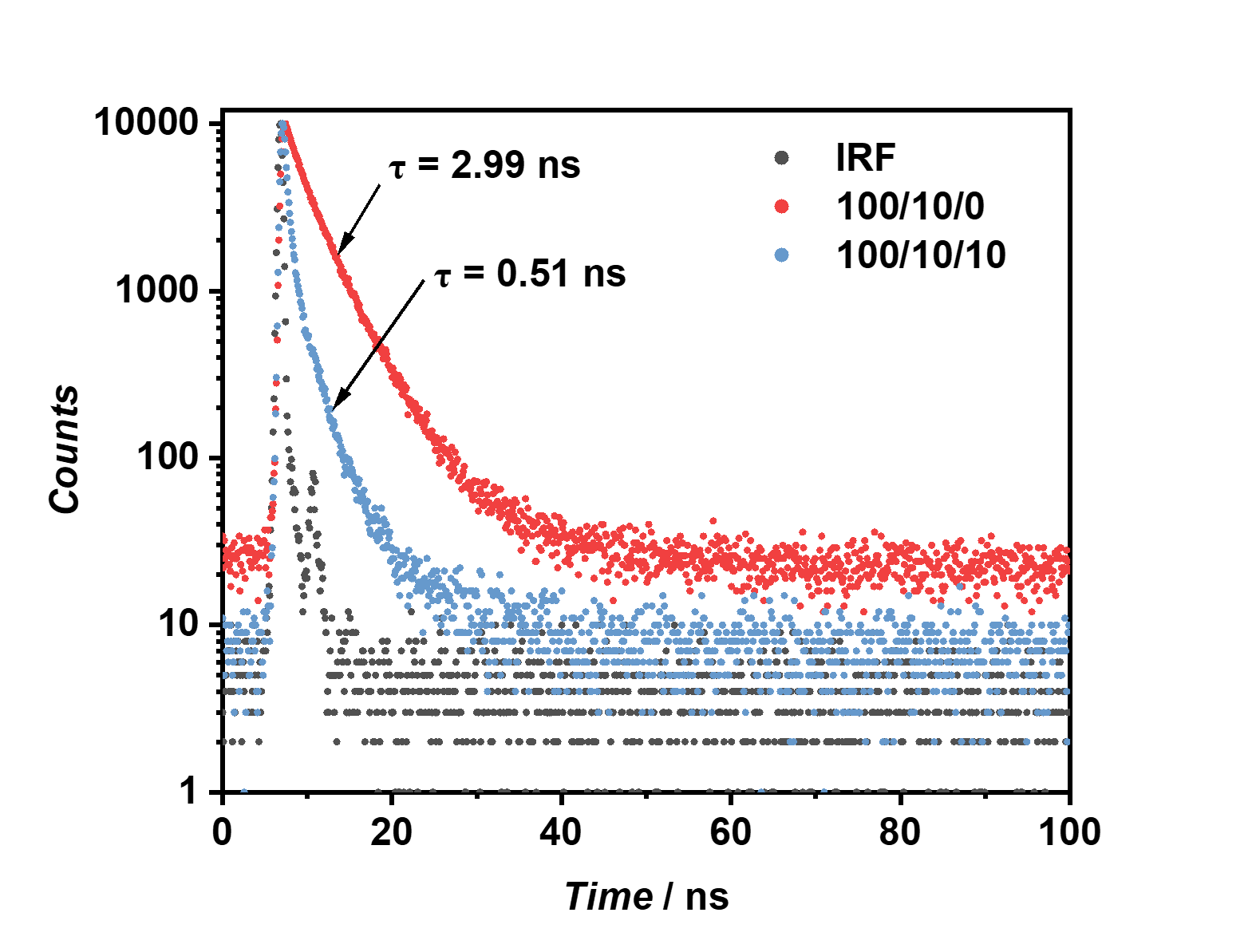


Figure S10 Time-resolved fluorescence spectra of **1/2** (100/10) and **1**/**2/3** (100/10/10)
 monitored at 480 nm ([**1**]=10 μM).


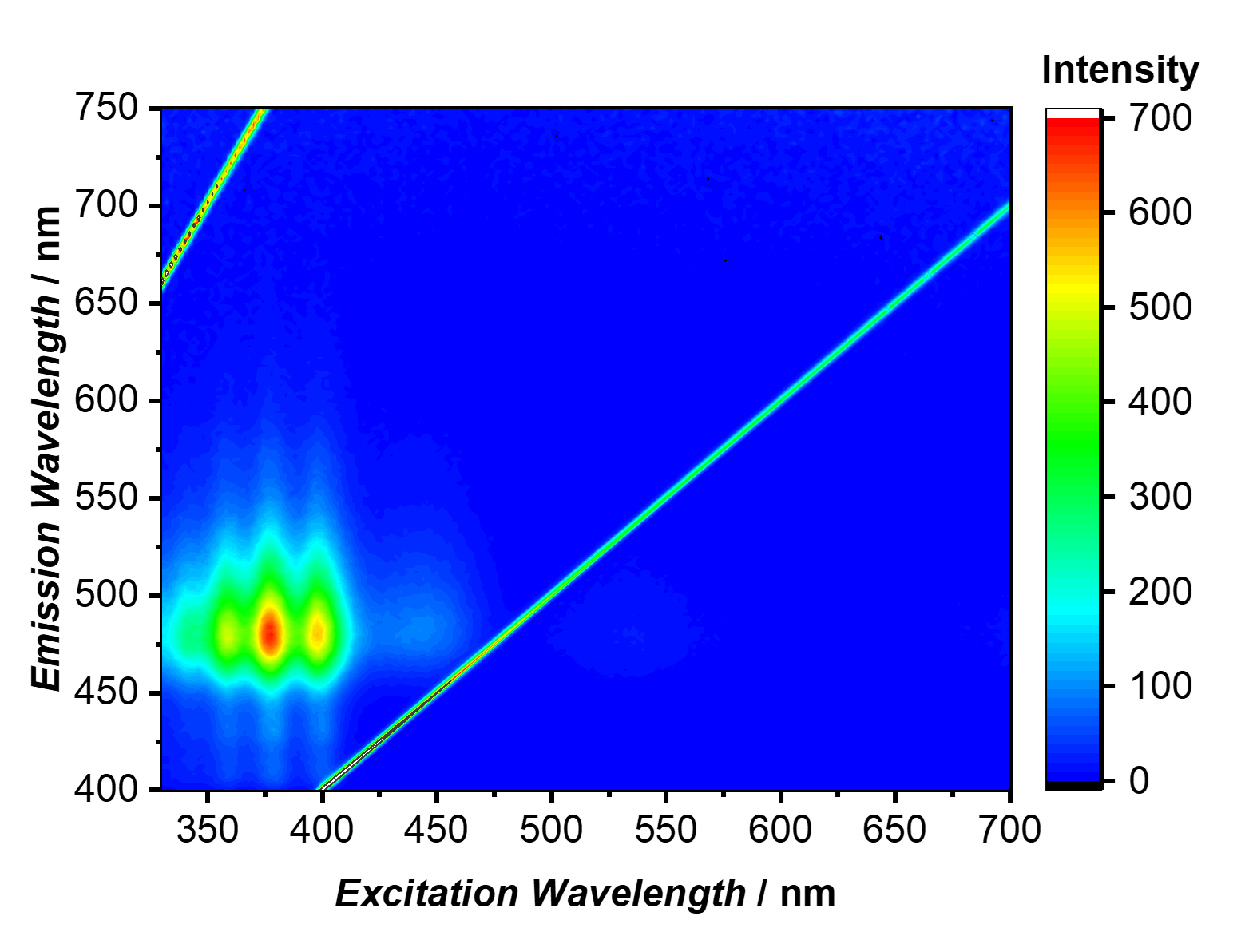


Figure S11 Two-dimensional excitation spectra of **1**/**2** (100/10).


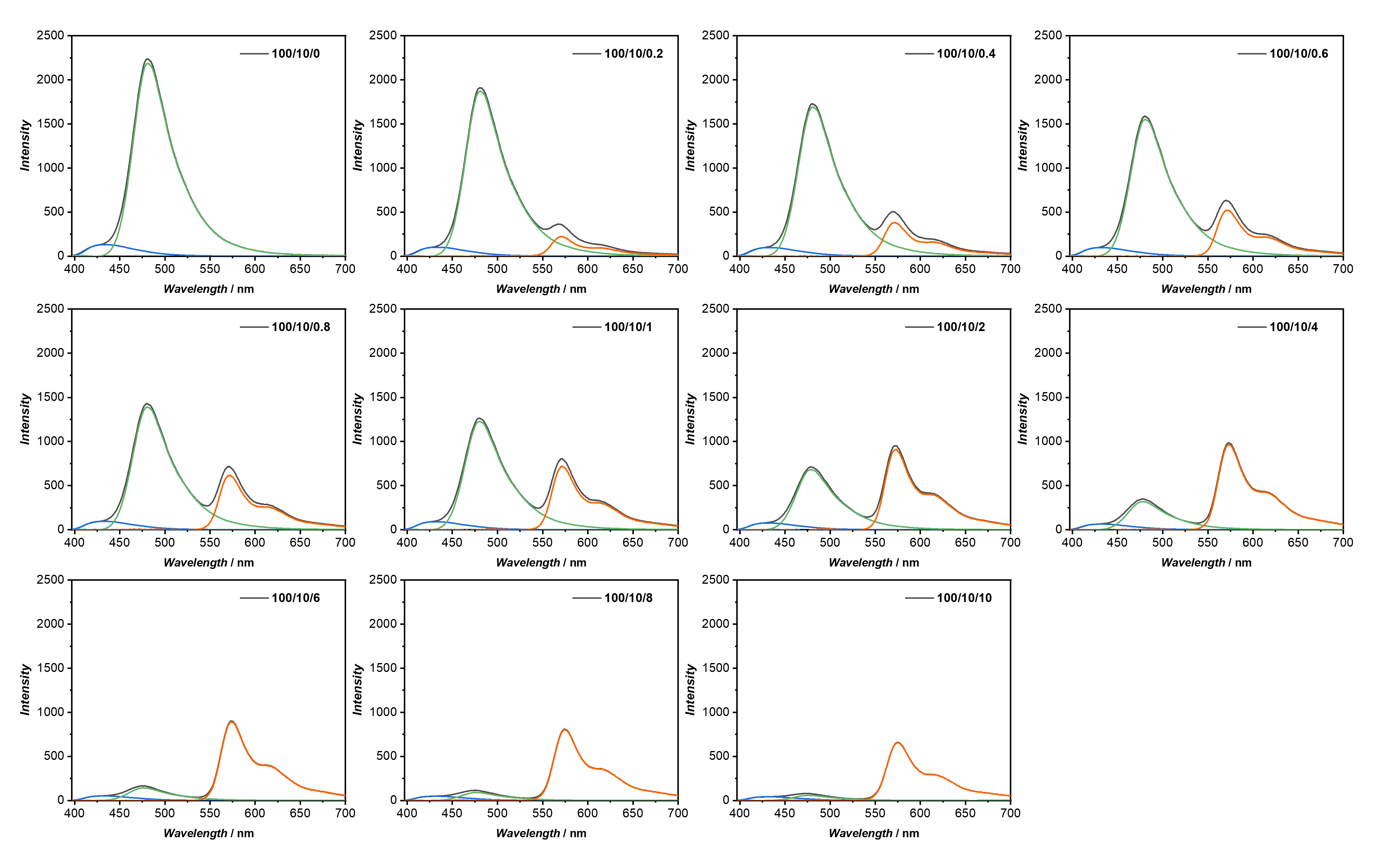


Figure S12 Deconvolution of emission spectra of **1**/**2/3** (grey) into emission bands of DPA (blue), Cou343 (green), and Cy3 (orange).


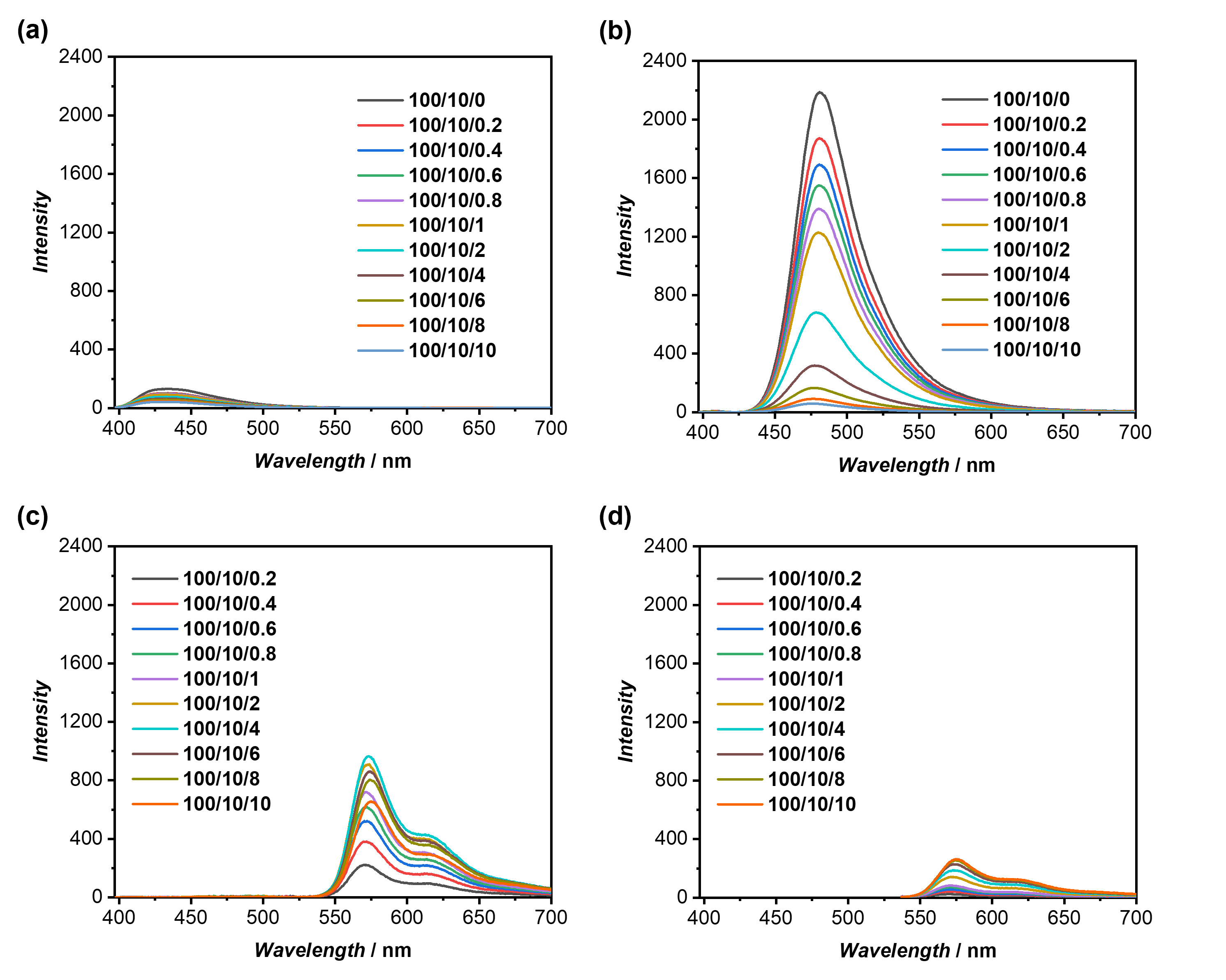


Figure S13 Deconvoluted emission spectra of DPA (a), Cou343 (b), and Cy3 (c); emission spectra of Cy3 when excited at 517 nm (d).


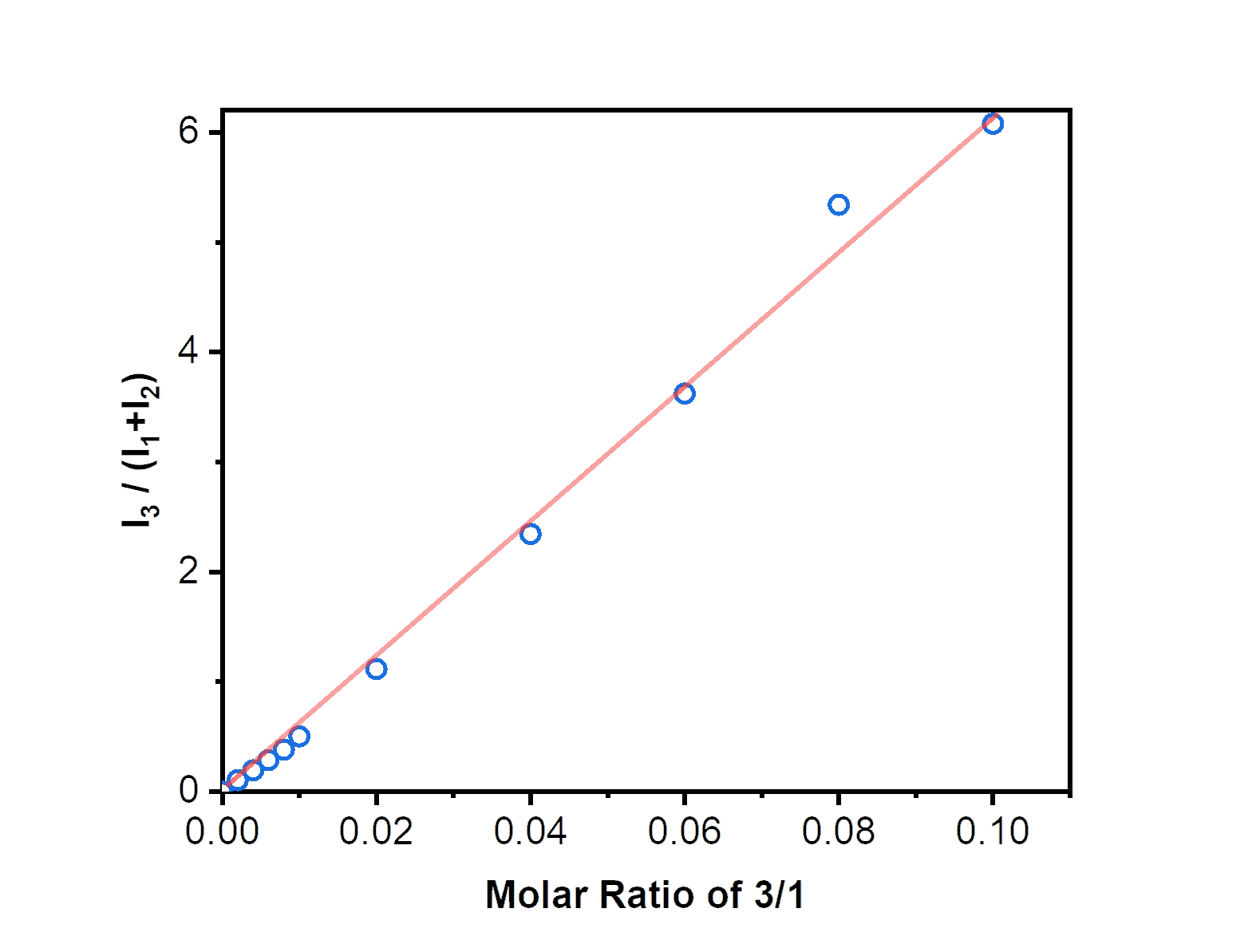


Figure S14 Ratiometric plot of ***I*_3_**/(***I*_1+_*I*_2_**).

*Calculation of energy transfer efficiency (Φ_ET_) and antenna effect (AE):* The *Φ*_ET_ and *AE* values were calculated using the equations shown below:

*Φ*_ET_ = 1-*I*_Cou343(100/10/0)_ / *I*_Cou343_

*AE* = *I*_Cy3_ / *I*_Cy3,direct_

Where *I*_Cy3,direct_ is the integral of the emission spectrum of Cy3 when excited at 517 nm (Figure S13d).

Table S4 The intensities of DPA (*I*_DPA_), Cou343 (*I*_DPA_), Cy3 (*I*_Cy3_), overall (*I*_total_) emission and calculated *Φ*_ET_ and *AE* values.

| Ratio | *I*_total_ | *I*_DPA_ | *I*_Cou343_ | *I*_Cy3_ | *Φ*_ET_ | *I*_Cy3,direct_ | *AE* |
| --- | --- | --- | --- | --- | --- | --- | --- |
| 100/10/0 | 139235 | 8913 | 130322 | 0 | - | - | - |
| 100/10/0.2 | 130666 | 6921 | 111572 | 12526 | 0.144 | 1770 | 7.1 |
| 100/10/0.4 | 128466 | 6650 | 100809 | 21006 | 0.226 | 2767 | 7.6 |
| 100/10/0.6 | 127489 | 6600 | 92400 | 28488 | 0.291 | 3577 | 8.0 |
| 100/10/0.8 | 123582 | 6491 | 82839 | 34252 | 0.364 | 4460 | 7.7 |
| 100/10/1 | 119324 | 6208 | 73175 | 39941 | 0.439 | 5069 | 7.9 |
| 100/10/2 | 96914 | 5212 | 40602 | 51099 | 0.688 | 8487 | 6.0 |
| 100/10/4 | 78212 | 4400 | 18982 | 54830 | 0.854 | 11453 | 4.8 |
| 100/10/6 | 63385 | 3871 | 9845 | 49669 | 0.924 | 13906 | 3.6 |
| 100/10/8 | 55321 | 3305 | 5421 | 46595 | 0.958 | 15378 | 3.0 |
| 100/10/10 | 44804 | 2869 | 3462 | 38473 | 0.973 | 15931 | 2.4 |


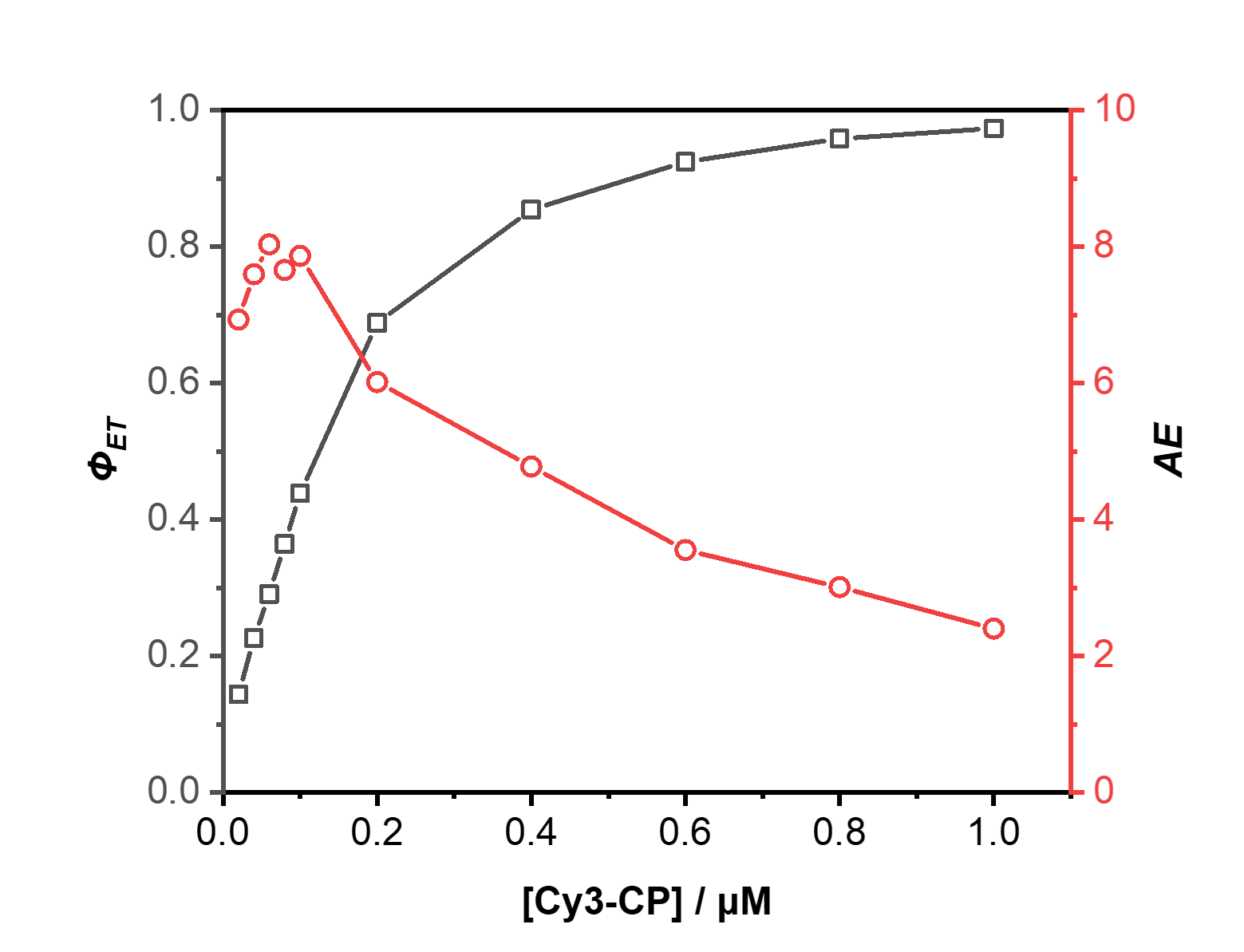


Figure S15 *Φ*_ET_ and *AE* values at different **1/2/3** molar ratios.


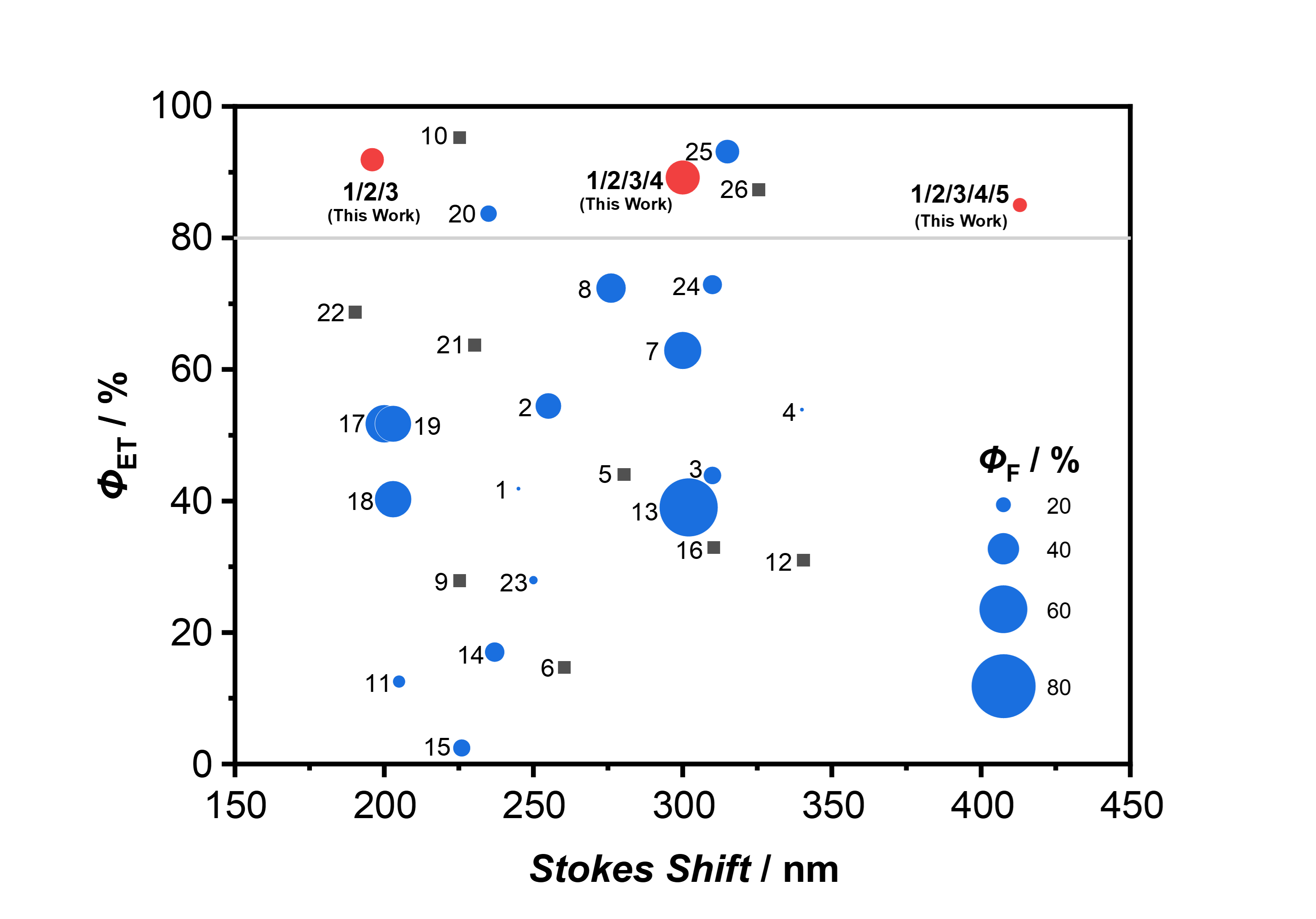


Figure S16 Comparison of performances of the artificial LHSs. (The size of the plots represents the level of *Φ*_F_. Plots represented by square symbols indicate that the *Φ*_F_ values are not reported.)

Table S5 Summary of photophysical properties of cascade artificial LHSs reported in literature.

| **No.** | **ALHS D-A_1_-A_2_** | ***Φ*_ET_**  **%** | ***AE*** | ***Φ*_F_**  **%** | **Stokes Shift**  **nm** | **D/A Ratio** | **Reference** |
| --- | --- | --- | --- | --- | --- | --- | --- |
| 1 | WP5/TPEDA-ESY-NiR | 74.4  56.3 | 3.5 | 5.0 | 245 | 100/0.5/0.5 | *Angew. Chem. Int. Ed.* **2020**, *59*, 10095. |
| 2 | WP5/BPT-DBT-NiR | 60.9  89.4 | 20.1 | 31.6 | 255 | 100/0.29/0.29 | *J. Mater. Chem. A* **2020**, *8*, 9590. |
| 3 | m-TPEWP5-MC-G-NiB | 70.2  62.5 | - | 22.1 | 310 | 100/400/10 | *Mater. Chem. Front.* **2023**, *7*, 2484. |
| 4 | Py-TPE/WP5-SR101-AlPcS_4_ | 64.0  84.2 | 11.6 | 5.0 | 340 | 100/0.67/0.075 | *Chem. Commun.* **2020**, *56*, 5949. |
| 5 | PPTA-WP5-EY-NIR | 67.0  66.0 | 7.2 | - | 280 | 100/5/2.5 | *ACS Appl. Mater. Interfaces* **2022**, *14*, 45734. |
| 6 | PPTA/BSC4-EY-SR101 | 36.6  40.8 | 20.3 | - | 260 | 100/0.5/0.25 | *Soft Matter* **2021**, *17*, 9871. |
| 7 | WP5/G-ESY-NiR | 79.0  79.6 | 7.8 | 45.5 | 300 | 100/1/0.4 | *Dyes Pigm.* **2023**, *210*, 110958. |
| 8 | WP5/G-ESY-SR101 | 77.0  94.0 | 13 | 36.4 | 276 | 100/2/0.6 | *Dyes Pigm.* **2023**, *215*, 111289. |
| 9 | H-GG-GY | 96.0  29.3 | 27.0 | - | 225 | 100/100/1 | *J. Mater. Chem. A* **2022**, *10*, 11332. |
| 10 |  | 96.0  99.3 | 4.0 | - | 225 | 100/100/50 |  |
| 11 | PPTA/CB[7]-EY-SR101 | 38.0  33.0 | 20.1 | 15.4 | 205 | 100/1/0.5 | *Dyes Pigm.* **2022**, *197*, 109895. |
| 12 | SOF-DBT-SR101 | 70.2  44.5 | 1.8 | - | 340 | 100/1/10 | *J. Mater. Chem. A* **2023**, *11*, 2627. |
| 13 | Dim/tQ[14]-ESY-NiR | 42.7  91.5 | 11.0 | 71.3 | 302 | 100/0.5/1 | *Chem. Eng. J.* **2022**, *446*, 136954. |
| 14 | M1-ESY-SR101 | 28.6  59.5 | 8.9 | 24.1 | 237 | 100/1/0.4 | *J. Am. Chem. Soc.* **2021**, *143*, 1313. |
| 15 | MPy1-ESY-SR101 | 12.2  20.1 | 9.3 | 21.3 | 226 | 100/1/0.5 | *J. Mater. Chem. C* **2022**, *10*, 12332. |
| 16 | MAPbBr_3_@Zn(II)TCP-EYB-NiB | 74.6  44.4 | - | - | 310 | - | *Chem. Commun.* **2021**, *57*, 9434. |
| 17 | 3a-ESY-NiR | 80.1  64.6 | 22.6 | 46.2 | 200 | 100/10/50 | *Angew. Chem. Int. Ed.* **2022**, *61*, e202200715. |
| 18 | P-EY-NiR | 58.8  68.5 | 10.9 | 45.0 | 203 | - | *Chem. Mater.* **2022**, *34*, 9656 |
| 19 | P-Fl-NiR | 67.9  76.2 | 17.7 | 44.5 | 203 | - |  |
| 20 | PYR-NTI-Cy3 | 88.1  95.0 | 1.1 | 20.6 | 235 | 100/8/8 | *J. Am. Chem. Soc.* **2021**, *143*, 382. |
| 21 | CD1-CD2-EY | 84.0  76.0 | - | - | 230 | 100/6/6 | *ACS Nano* **2022**, *16*, 5, 8012. |
| 22 | CG-ThT-AO | 93.0  74.0 | 8.2 | - | 190 | 100/10/2 | *Angew. Chem. Int. Ed.* **2019**, *58*, 844. |
| 23 | PPTA/GP5A-EY-NiR | 38.9  71.9 | 13.5 | 10.9 | 250 | 100/0.4/0.7 | *Chin. Chem. Lett.* **2023**, *34*, 108081. |
| 24 | M-DBT-NDI | 87.4  83.4 | 47.6 | 23.9 | 310 | 100/1/0.5 | *Chem. Commun.* **2021**, *57*, 5782. |
| 25 | BDA-TPEM-NiR | 97.6  95.5 | 17.1 | 29.5 | 315 | 100/20/4 | *J. Am. Chem. Soc.* **2022**, *144*, 12, 5389. |
| 26 | σ-platinated (hetero)acenes **1**-**2**-**3** | 92.4  92.5 | 0.4 | - | 325 | 100/20/10 | *Nat. Commun.* **2022**, *13*, 3546. |
| 27 | **1**-**2**-**3** | 90.5  92.4 | 3.6 | 28.7 | 196 | 100/10/6 | Our work |
| 28 | **1**-**2**-**3**-**4** | 90.5  92.4  90.5 | 4.0 | 42.0 | 300 | 100/10/6/6 | Our work |
| 29 | **1**-**2**-**3**-**4**-**5** | 90.5  92.4  90.5  89.3 | 2.9 | 17.6 | 413 | 100/10/6/5/5 | Our work |

*Calculation of static and dynamic quenching:* The total fluorescence quenching efficiency (*η*_tot_) is the sum of dynamic quenching efficiency (*η*_dyn_) and static quenching efficiency (*η*_stat_). The static quenching and dynamic quenching values were calculated using the equations shown below:

*η*_dyn_ = 1- *τ*/*τ*_0_

*η*_tot_ = *Φ*_ET_ = 1-*I*_Cou343(100/10/0)_ / *I*_Cou343_

*η*_stat_ = *η*_tot_ - *η*_dyn_

**
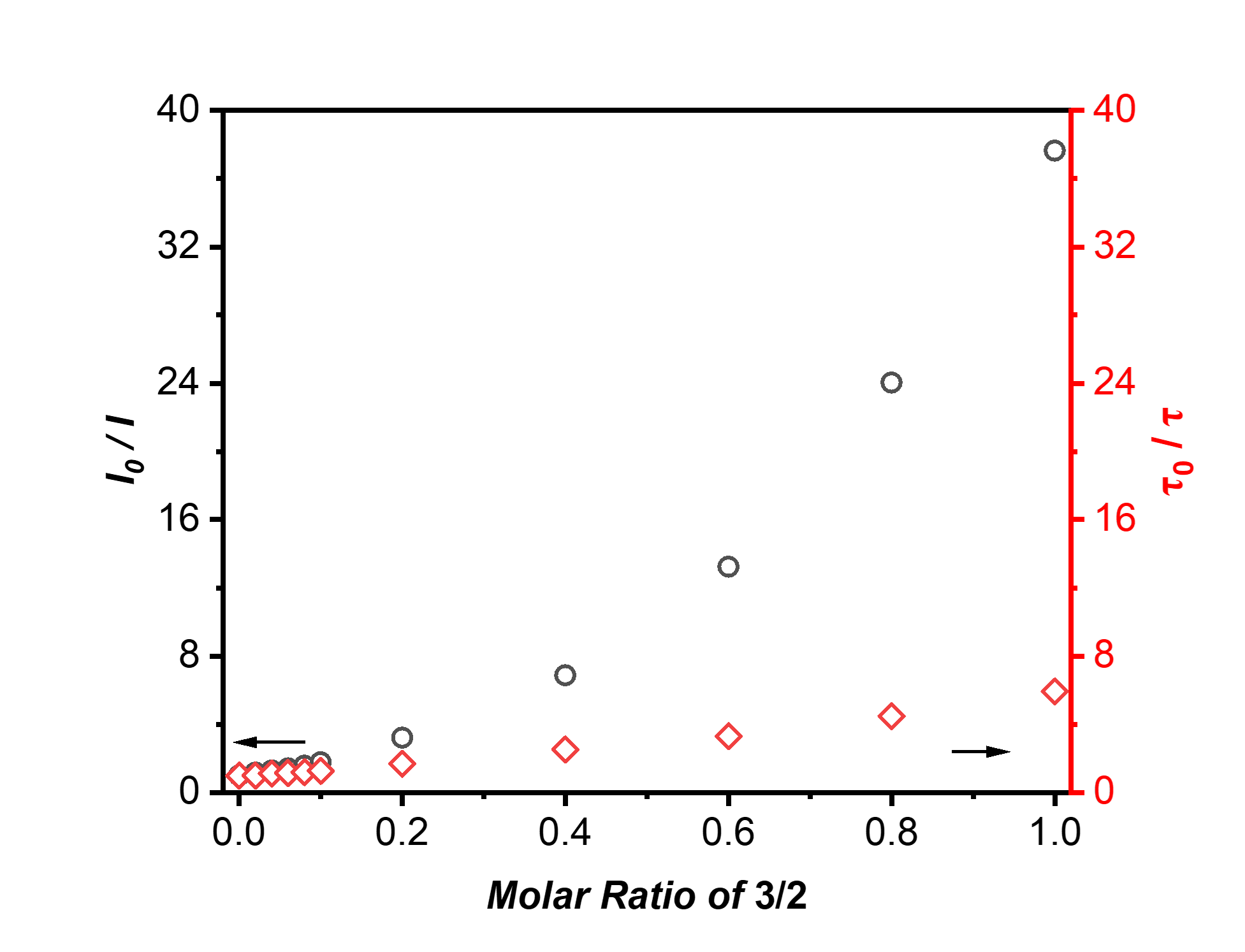
**

Figure S17 Stern-Volmer plots of the steady-state fluorescence intensity of **2** and the average fluorescence lifetime of **2**.

Table S6 Dynamic and static quenching of **1**/**2/3** upon varying the molar ratios of **1**/**2/3**.

| **1**/**2**/**3** | *τ*_avg_ / ns | *η*_tot_ | *η*_dyn_ | *η*_stat_ |
| --- | --- | --- | --- | --- |
| 100/10/0 | 2.99 | - | 0.0% | - |
| 100/10/0.4 | 2.68 | 22.6% | 10.4% | 12.3% |
| 100/10/0.6 | 2.62 | 29.1% | 12.4% | 16.7% |
| 100/10/0.8 | 2.50 | 36.4% | 16.4% | 20.0% |
| 100/10/1 | 2.37 | 43.9% | 20.7% | 23.1% |
| 100/10/2 | 1.78 | 68.8% | 40.5% | 28.4% |
| 100/10/4 | 1.19 | 85.4% | 60.2% | 25.2% |
| 100/10/6 | 0.91 | 92.4% | 69.6% | 22.9% |
| 100/10/8 | 0.67 | 95.8% | 77.6% | 18.2% |
| 100/10/10 | 0.51 | 97.3% | 82.9% | 14.4% |

*Determination of the number of donors (n) that can be quenched by a single acceptor*: Through non-linear fitting of the emission intensities of **2** (*I*_F_) against the concentration of **3** (*c*_A_), *c*_0_ was calculated to be 1.44×10^-7^ M, which gave *n* = *c*(**2**) /*c*_0_ =6.9 (Figure S18a).

*Calculation of the second-order exciton migration rate constant*: By plotting the reciprocal of fluorescent lifetimes (1/*τ*) of **2** versus the concentration of **3**, the second-order rate constant for the exciton migration process is equal to the slope of the linearly fitted line (Figure S18b).


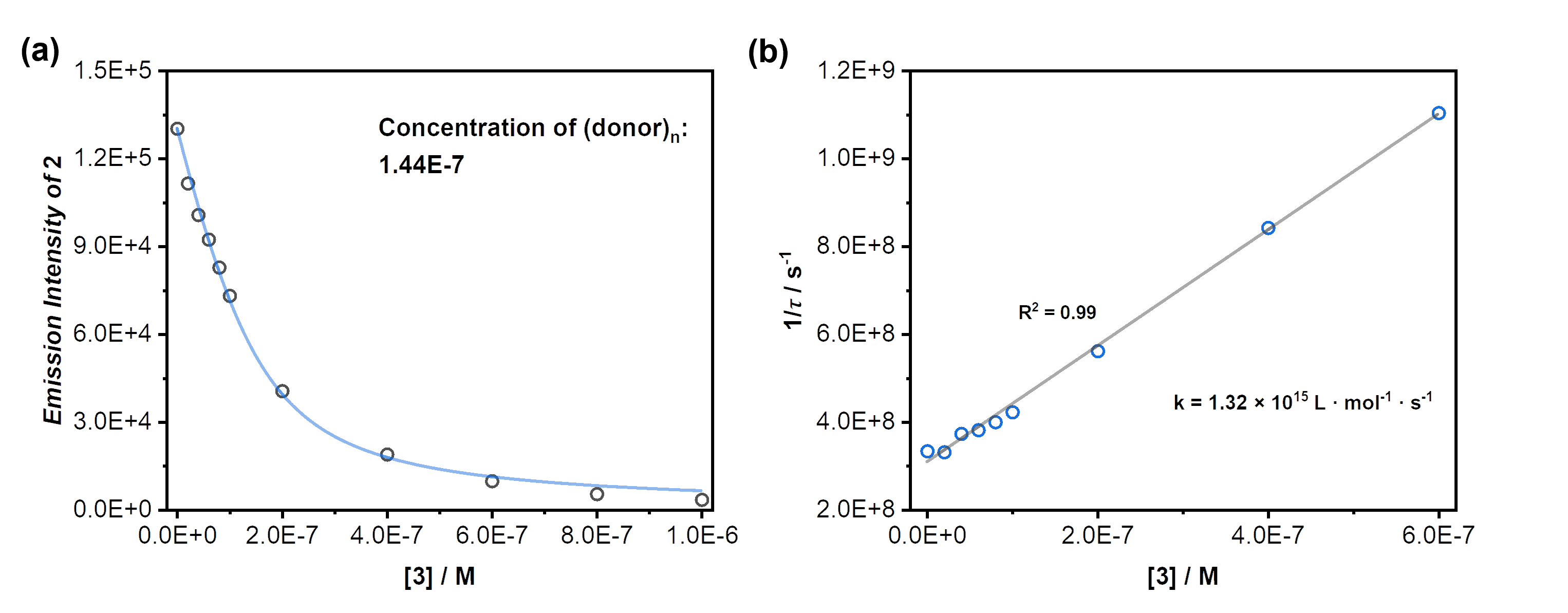


Figure S18 (a) Non-linear fitting of the emission intensities of **2** versus the concentration of **3**; (f) Plots of 1/*τ* values of **2** versus the concentration of **3**.

*Calculation of the k_ET_ values*

*a. Calculation of the k_ET_ values of the two-component systems (****1/2****,* ***1/3****,* ***1/4****)*

*k_ET_* represents energy transfer rate constant. It can be determined via the equations shown below:

$$k_{ET}=\left( \frac{1}{\tau_{D}} \right)\times\left( \frac{R_{0}}{d} \right)^{6}$$

$$\Phi_{ET}=\frac{{R_{0}}^{6}}{({R_{0}}^{6}+d^{6})}$$

And we can get this equation by means of two above equations:

$$k_{ET}=\left( \frac{1}{\tau_{D}} \right)\times(\frac{\Phi_{ET}}{1-\Phi_{ET}})$$

In these equations, $\tau_{D}$ is the fluorescence lifetime of donor **1** in the absence of acceptors. $d$ stands for the average distance of D/A pair. $R_{0}$ stands for the Fröster radius of D/A pair.

Therefore, to obtain the *k_ET_* values, two additional binary systems of **1**/**3** and **1**/**4** were investigated, as shown in Figure S19. The calculated *k_ET_* values are listed in Table S7.


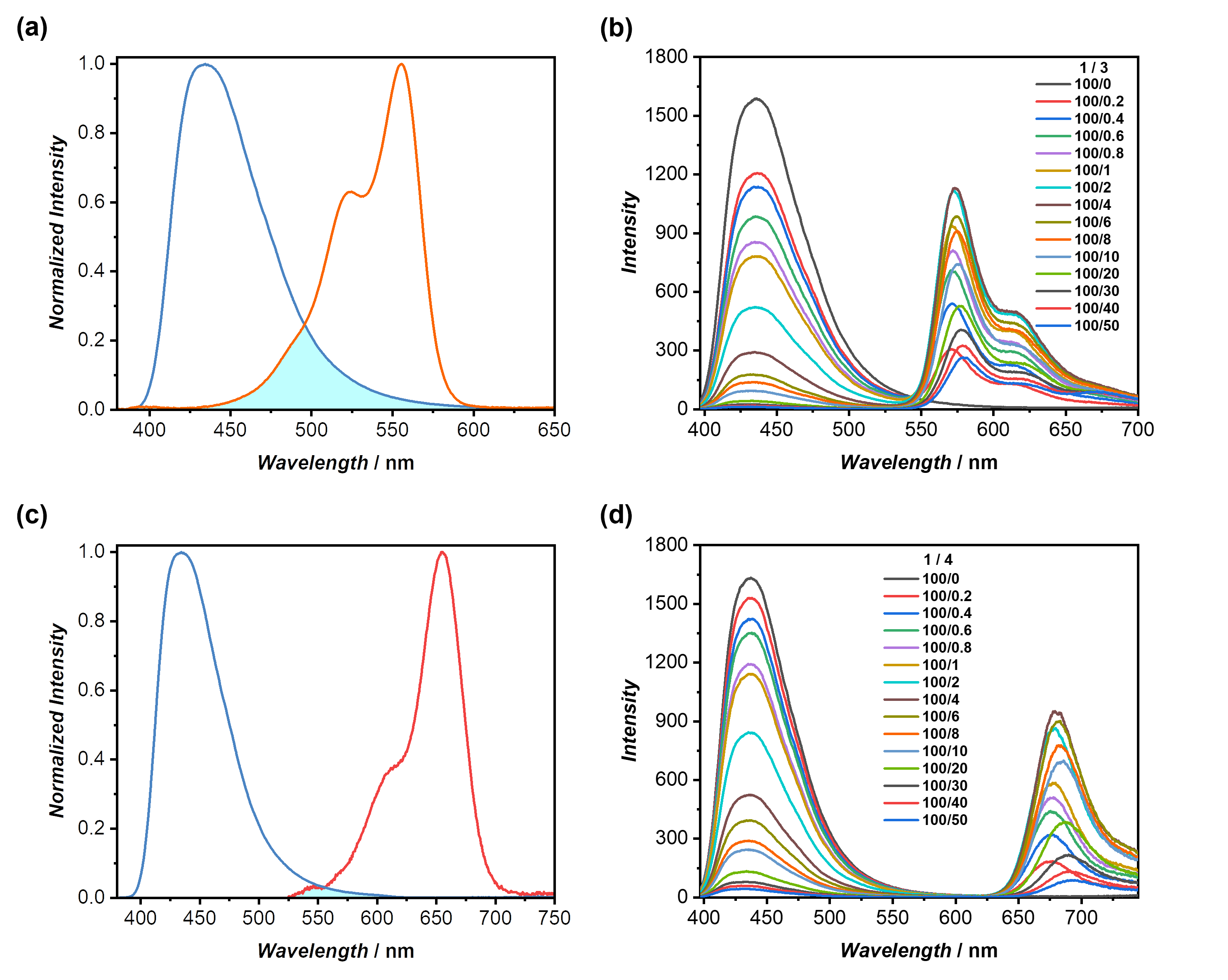


Figure S19 (a) Normalized fluorescence spectrum of DPA-CP-PEG (**1**, blue trace) and absorption spectrum of Cy3-CP (**3**, orange trace); (b) Fluorescence spectra of DPA-CP-PEG with different concentrations of Cy3-CP (*λ*_ex_ = 377 nm); (c) Normalized fluorescence spectrum of DPA-CP-PEG (**1**, blue trace) and absorption spectrum of Cy5-CP (**4**, red trace); (d) Fluorescence spectra of DPA-CP-PEG with different concentrations of Cy5-CP (*λ*_ex_ = 377 nm).

Table S7 Calculated *Φ*_ET_ and *k*_ET_ values of the 3 binary systems.

|  | **1**/**2** | | **1**/**3** | | **1**/**4** | |
| --- | --- | --- | --- | --- | --- | --- |
| Ratio | *Φ*_ET_ | *k*_ET, 1/2_ / s^-1^ | *Φ*_ET_ | *k*_ET, 1/3_ / s^-1^ | *Φ*_ET_ | *k*_ET, 1/4_ / s^-1^ |
| 100/0.2 | 0.225 | 7.28×10^7^ | 0.241 | 7.96×10^7^ | 0.064 | 1.72×10^7^ |
| 100/0.4 | 0.322 | 1.19×10^8^ | 0.283 | 9.87×10^7^ | 0.130 | 3.72×10^7^ |
| 100/0.6 | 0.374 | 1.50×10^8^ | 0.377 | 1.52×10^8^ | 0.174 | 5.29×10^7^ |
| 100/0.8 | 0.440 | 1.97×10^8^ | 0.461 | 2.14×10^8^ | 0.271 | 9.30×10^7^ |
| 100/1 | 0.487 | 2.38×10^8^ | 0.507 | 2.57×10^8^ | 0.302 | 1.08×10^8^ |
| 100/2 | 0.658 | 4.82×10^8^ | 0.671 | 5.11×10^8^ | 0.485 | 2.36×10^8^ |
| 100/4 | 0.757 | 7.81×10^8^ | 0.817 | 1.12×10^9^ | 0.681 | 5.33×10^8^ |
| 100/6 | 0.838 | 1.29×10^9^ | 0.889 | 2.01×10^9^ | 0.760 | 7.94×10^8^ |
| 100/8 | 0.878 | 1.79×10^9^ | 0.913 | 2.63×10^9^ | 0.824 | 1.17×10^9^ |
| 100/10 | 0.905 | 2.39×10^9^ | 0.941 | 4.00×10^9^ | 0.852 | 1.44×10^9^ |
| 100/20 | 0.955 | 5.30×10^9^ | 0.974 | 9.28×10^9^ | 0.920 | 2.89×10^9^ |
| 100/30 | 0.972 | 8.82×10^9^ | 0.985 | 1.59×10^10^ | 0.952 | 5.02×10^9^ |
| 100/40 | 0.984 | 1.51×10^10^ | 0.989 | 2.30×10^10^ | 0.964 | 6.78×10^9^ |
| 100/50 | 0.988 | 2.11×10^10^ | 0.992 | 3.17×10^10^ | 0.974 | 9.42×10^9^ |

*b. Calculation of the spectral overlap of the two-component systems (****1/2****,* ***1/3****,* ***1/4****)*

The spectral overlap integral $J(\lambda)$ between D/A pairs was calculated based on the equation below:

$$J\left( \lambda\right)=\frac{\left[ \int_{0}^{\infty} F_{D}\left( \lambda\right)\varepsilon_{A}(\lambda)\lambda^{4}d\lambda\right]}{\left[ \int_{0}^{\infty} F_{D}\left( \lambda\right)d\lambda\right]}$$

In this equation, $F_{D}\left( \lambda\right)$ is the fluorescence emission spectrum of donor, while $\varepsilon_{A}(\lambda)$ is the molar absorption coefficient of acceptor. $\lambda$ denotes the wavelength of absorption or emission spectrum. $F_{D}\left( \lambda\right)$ is a dimensionless term in the formula and the value of denominator is normalized to 1 before calculation.

*c. Calculation of the k_ET_ values of the ternary system* ***1/2/3***

*k_ET,1/2_* and *k_ET,1/3_* were calculated using the equations below:

$$\Phi_{ET,1/2+1/3}=\frac{k_{ET,1/2}+k_{ET,1/3}}{1/\tau_{D}{+k}_{ET,1/2}+k_{ET,1/3}} {(\tau}_{D}=4.00 ns)$$

$$\Phi_{ET,1/2+1/3}=1-\frac{I_{DPA}}{I_{DPA(100/10/0)}}\times(1-\Phi_{ET,1/2(100/10)}) \Phi_{ET,1/2(100/10)}=0.905$$

Taking **1/2/3** = **100/10/6** as an instance (using the data from Table S4 and Table S7):

$$\frac{k_{ET,1/2}}{k_{ET,1/3}}=\frac{2.39\times{10}^{9}}{2.01\times{10}^{9}}=1.189$$

$$\Phi_{ET,1/2+1/3}=\frac{k_{ET,1/2}+k_{ET,1/3}}{1/\tau_{D}{+k}_{ET,1/2}+k_{ET,1/3}}=0.9588$$

Which gives *k_ET,1/2_* and *k_ET,1/3_* as 3.16×10^9^ s^-1^ and 2.66×10^9^ s^-1^, respectively.

*k_ET,2/3_* was calculated using the equation below:

$$\Phi_{ET,2/3}=\frac{k_{ET,2/3}}{1/\tau_{1/2}{+k}_{ET,2/3}} {(\tau}_{1/2}=2.99 ns)$$

*d. Calculation of the Ф_ET_ values of the ternary system* ***1/2/3***

$$\Phi_{ET,1/2}=\frac{k_{ET,1/2}}{1/\tau_{D}{+k}_{ET,1/2}+k_{ET,1/3}}$$

$$\Phi_{ET,1/3}=\frac{k_{ET,1/3}}{1/\tau_{D}{+k}_{ET,1/2}+k_{ET,1/3}}$$

$$\Phi_{ET,overall}=\Phi_{ET,1/2}\times\Phi_{ET,2/3}+\Phi_{ET,1/3}$$

Table S8 Calculated *Φ*_ET_ and *k*_ET_ values of the **1**/**2**/**3** ternary system.

| Ratio | *k*_ET, 1/2_ / s^-1^ | *k*_ET, 1/3_ / s^-1^ | *k*_ET, 2/3_ / s^-1^ | *Ф*_ET,1/2_ | *Ф*_ET,1/3_ | *Ф*_ET,2/3_ | *Ф*_ET,overall_ |
| --- | --- | --- | --- | --- | --- | --- | --- |
| 100/10/0.2 | 3.05×10^9^ | 1.01×10^8^ | 5.61×10^7^ | 0.897 | 0.030 | 0.144 | 0.159 |
| 100/10/0.4 | 3.16×10^9^ | 1.30×10^8^ | 9.78×10^7^ | 0.892 | 0.037 | 0.227 | 0.239 |
| 100/10/0.6 | 3.11×10^9^ | 1.98×10^8^ | 1.37×10^8^ | 0.874 | 0.056 | 0.291 | 0.310 |
| 100/10/0.8 | 3.09×10^9^ | 2.77×10^8^ | 1.91×10^8^ | 0.854 | 0.077 | 0.364 | 0.388 |
| 100/10/1 | 3.19×10^9^ | 3.44×10^8^ | 2.61×10^8^ | 0.843 | 0.091 | 0.439 | 0.460 |
| 100/10/2 | 3.51×10^9^ | 7.51×10^8^ | 7.38×10^8^ | 0.778 | 0.167 | 0.688 | 0.702 |
| 100/10/4 | 3.47×10^9^ | 1.63×10^9^ | 1.96×10^9^ | 0.649 | 0.305 | 0.854 | 0.859 |
| 100/10/6 | 3.16×10^9^ | 2.66×10^9^ | 4.09×10^9^ | 0.521 | 0.438 | 0.925 | 0.919 |
| 100/10/8 | 3.27×10^9^ | 3.60×10^9^ | 7.69×10^9^ | 0.459 | 0.506 | 0.958 | 0.946 |
| 100/10/10 | 2.97×10^9^ | 4.97×10^9^ | 1.22×10^10^ | 0.363 | 0.607 | 0.973 | 0.960 |

**S5. Quaternary LHS with Three-Step Sequential Energy Transfer**


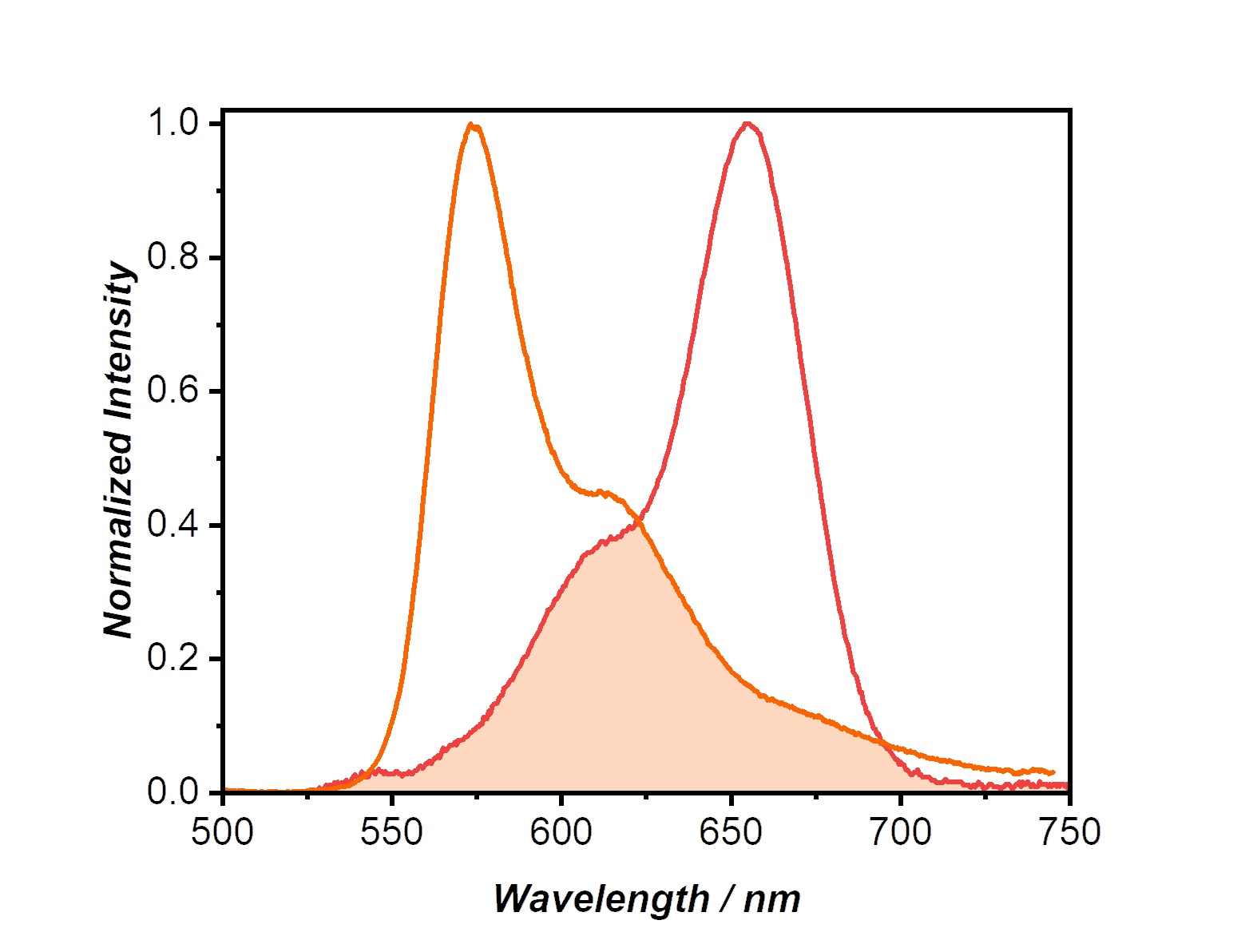


Figure S20 Spectral overlap of the normalized fluorescence spectrum of **3** (orange) and absorption spectrum of **4** (red).


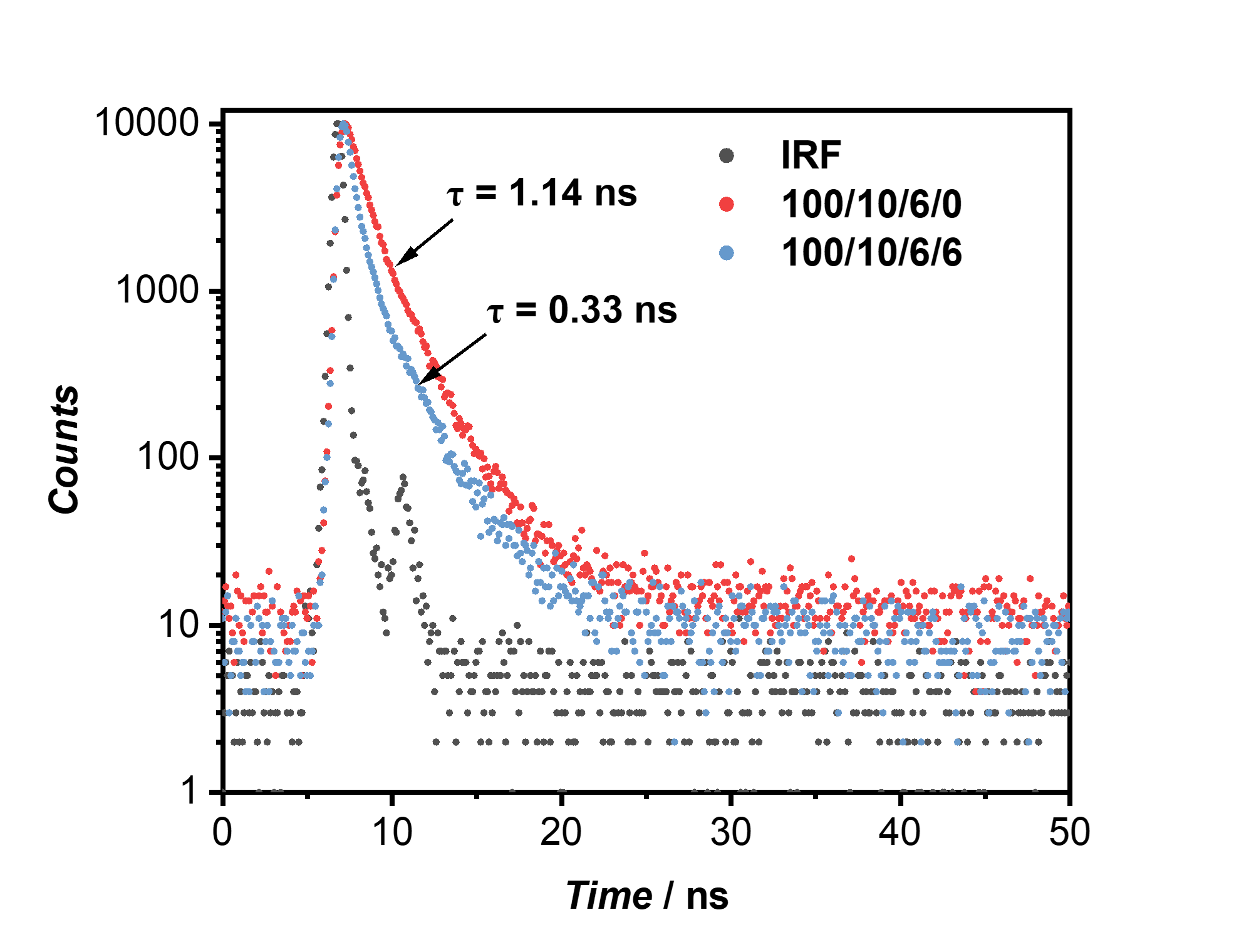


Figure S21 Time-resolved fluorescence spectra of **1/2/3** (100/10/6) and **1**/**2/3/4** (100/10/6/6) monitored at 575 nm ([**1**]=10 μM).


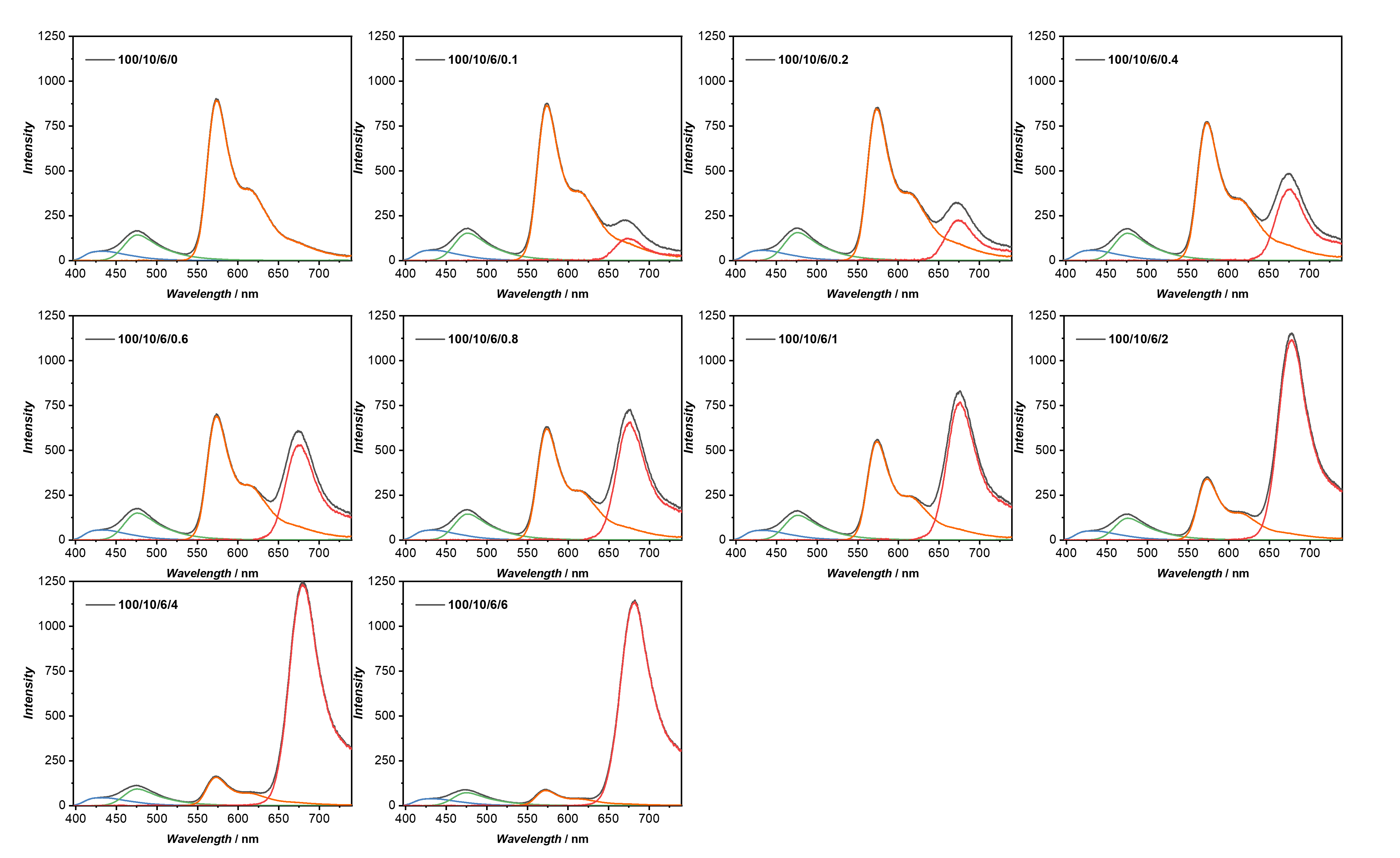


Figure S22 Deconvolution of emission spectra of **1**/**2/3/4** (grey) into emission bands of DPA (blue), Cou343 (green), Cy3 (orange), and Cy5 (red).


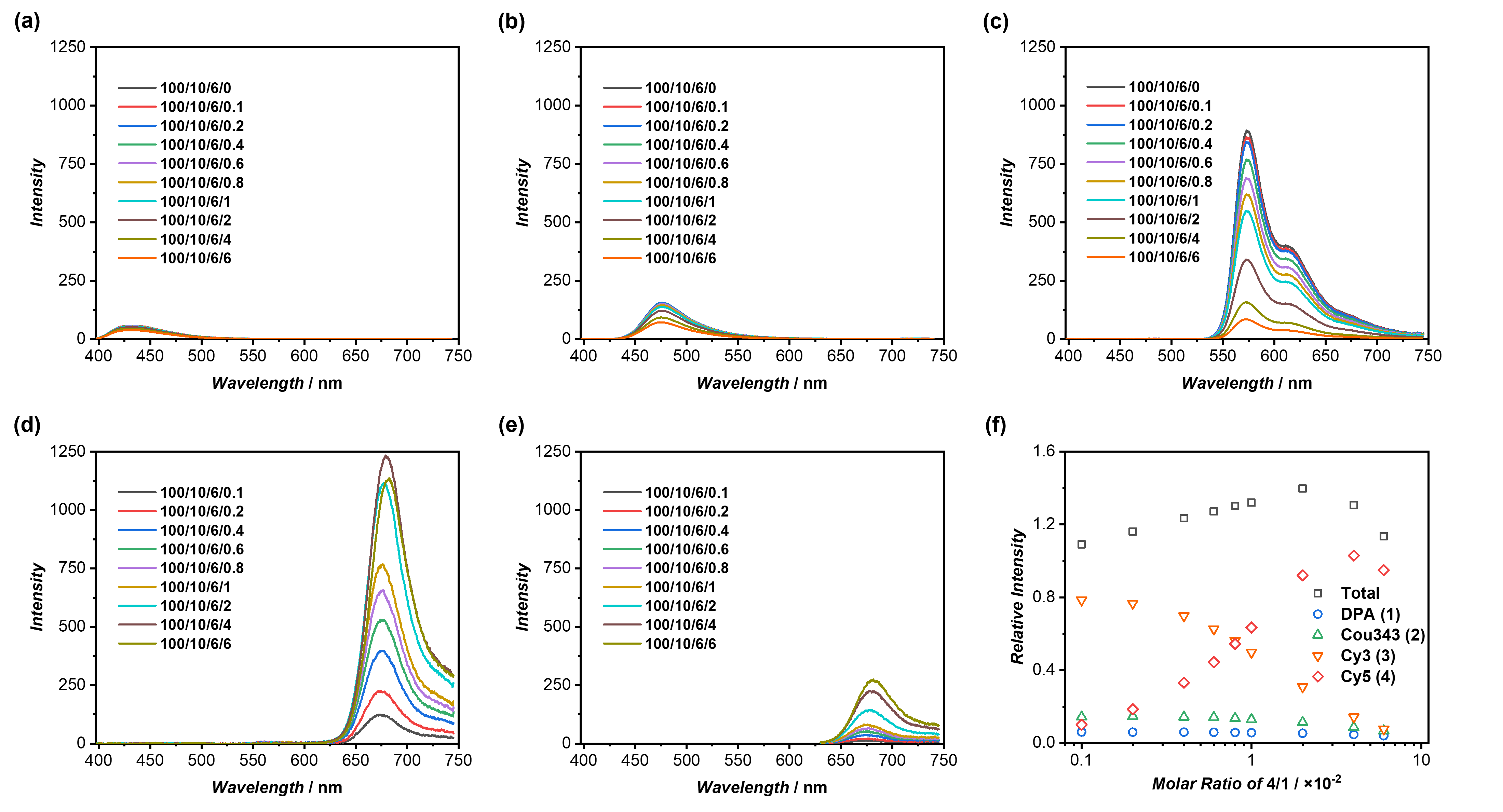


Figure S23 Deconvoluted emission spectra of DPA (a), Cou343 (b), Cy3 (c), and Cy5 (d); emission spectra of Cy5 when excited at 610 nm (e); Ratiometric plot of the total, DPA, Cou343, Cy3, and Cy5 emission intensities (f).

*Calculation of energy transfer efficiency (Φ_ET_) and antenna effect (AE):* The *Φ*_ET_ and *AE* values were calculated using the equations shown below:

*Φ*_ET_ = 1-*I*_Cy3(100/10/6/0)_ / *I*_Cy3_

*AE* = *I*_Cy5_ / *I*_Cy5,direct_

Where *I*_Cy5,direct_ is the integral of the emission spectrum of Cy5 when excited at 610 nm (Figure S23e).

Table S9 The intensities of DPA (*I*_DPA_), Cou343 (*I*_DPA_), Cy3 (*I*_Cy3_), Cy5 (*I*_Cy5_), overall (*I*_total_) emission and calculated *Φ*_ET_ and *AE* values.

| Ratio | *I*_total_ | *I*_DPA_ | *I*_Cou343_ | *I*_Cy3_ | *I*_Cy5_ | *Φ*_ET_ | *I*_Cy5,direct_ | *AE* |
| --- | --- | --- | --- | --- | --- | --- | --- | --- |
| 100/10/6/0 | 64436 | 3553 | 8670 | 52213 | 0 | 0 | 0 | 0 |
| 100/10/6/0.1 | 70323 | 3943 | 9284 | 50586 | 6510 | 0.031 | 862 | 7.6 |
| 100/10/6/0.2 | 74778 | 3873 | 9481 | 49427 | 11997 | 0.053 | 1324 | 9.1 |
| 100/10/6/0.4 | 79507 | 3895 | 9252 | 44985 | 21375 | 0.138 | 2205 | 9.7 |
| 100/10/6/0.6 | 81948 | 3835 | 9136 | 40351 | 28624 | 0.227 | 2989 | 9.6 |
| 100/10/6/0.8 | 83908 | 3759 | 8816 | 36263 | 35069 | 0.305 | 3741 | 9.4 |
| 100/10/6/1 | 85097 | 3688 | 8372 | 32113 | 40923 | 0.385 | 4556 | 9.0 |
| 100/10/6/2 | 90091 | 3490 | 7384 | 19897 | 59319 | 0.619 | 7991 | 7.4 |
| 100/10/6/4 | 84223 | 3032 | 5639 | 9226 | 66326 | 0.823 | 12487 | 5.3 |
| 100/10/6/6 | 73160 | 2627 | 4384 | 4955 | 61193 | 0.905 | 15117 | 4.0 |


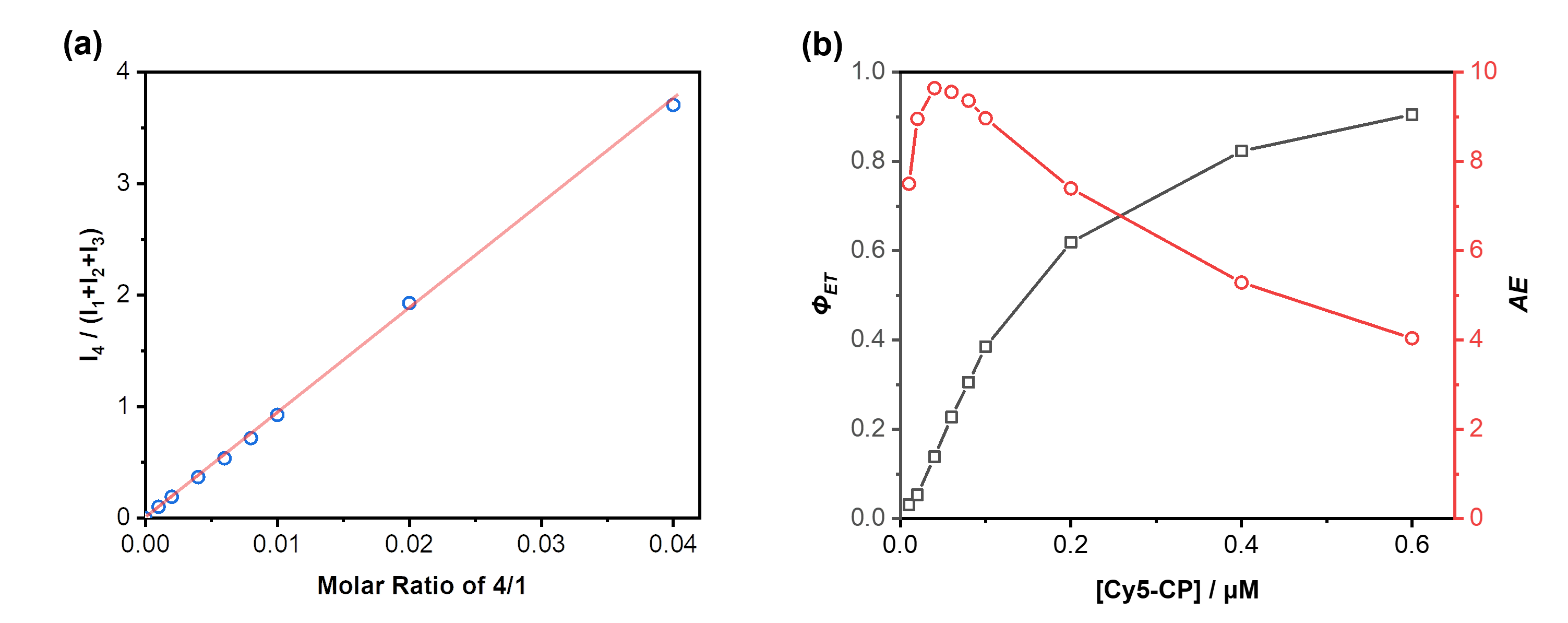


Figure S24 (a) Ratiometric plot of ***I*_4_**/(***I*_1+_*I*_2+_*I*_3_**); (b) *Φ*_ET_ and *AE* values
 at different **1/2/3/4** molar ratios.

*Determination of the number of donors (n) that can be quenched by a single acceptor*: Through non-linear fitting of the emission intensities of **3** (*I*_F_) against the concentration of **4** (*c*_A_), *c*_0_ was calculated to be 1.96×10^-7^ M, which gave *n* = *c*(**3**) /*c*_0_ =3.1 (Figure S25a).

*Calculation of the second-order exciton migration rate constant*: By plotting the reciprocal of fluorescent lifetimes (1/*τ*) of **3** versus the concentration of **4**, the second-order rate constant for the exciton migration process is equal to the slope of the linearly fitted line (Figure S25b).


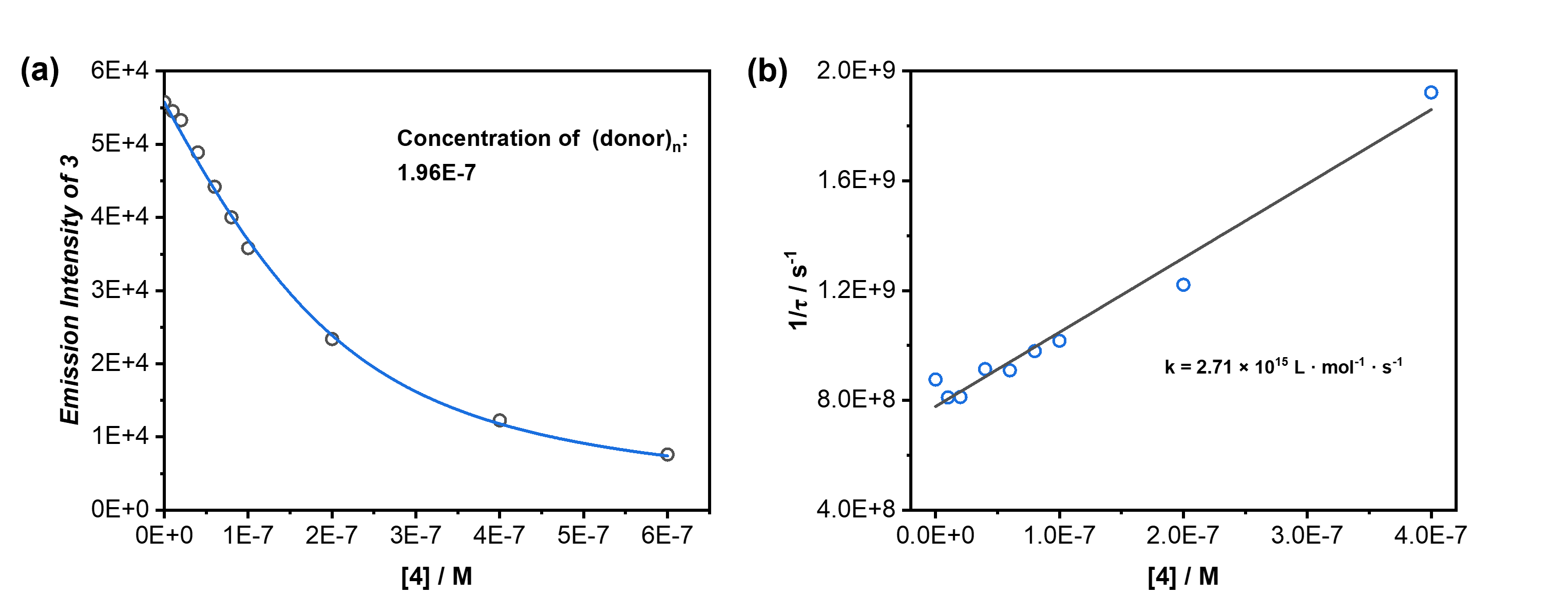


Figure S25 (a) Non-linear fitting of the emission intensities of **2** versus the concentration of **3**; (f) Plots of 1/*τ* values of **2** versus the concentration of **3**.

*Calculation of the k_ET_ and Ф_ET_ values of the quaternary system* ***1/2/3/4***

*a. Calculation of k*_ET, 1/2_, *k*_ET, 1/3_ and *k*_ET, 1/4_

*k*_ET, 1/2_, *k*_ET, 1/3_ and *k*_ET, 1/4_ were calculated using the equations below:

$$\Phi_{ET,1/2+1/{3+1/4}}=\frac{k_{ET,1/2}+k_{ET,1/3}+k_{ET,1/4}}{1/\tau_{1}{+k}_{ET,1/2}+k_{ET,1/3}+k_{ET,1/4}} {(\tau}_{1}=4.00 ns)$$

$$\Phi_{ET,1/2+1/3}=1-\frac{I_{DPA}}{I_{DPA(100/10/6/0)}}\times(1-\Phi_{ET,1/{2+1/3}(100/10/6)}) \Phi_{ET,1/{2+1/3}(100/10/6)}=0.959$$

Taking **1/2/3/4** = **100/10/6/6** as an instance (using the data from Table S7 and Table S9):

$$\frac{k_{ET,1/2}}{k_{ET,1/3}}=\frac{2.39\times{10}^{9}}{2.01\times{10}^{9}}=1.189$$

$$\frac{k_{ET,1/2}}{k_{ET,1/4}}=\frac{2.39\times{10}^{9}}{7.94\times{10}^{8}}=3.010$$

$$\Phi_{ET,1/2+1/{3+1/4}}=\frac{k_{ET,1/2}+k_{ET,1/3}+k_{ET,1/4}}{1/\tau_{1}{+k}_{ET,1/2}+k_{ET,1/3}+k_{ET,1/4}}=0.9695$$

Which gives *k_ET,1/2_*, *k_ET,1/3_* and *k_ET,1/4_* as 3.66×10^9^ s^-1^, 3.08×10^9^ s^-1^_,_ and 1.22×10^9^ s^-1^, respectively.

*b. Calculation of k*_ET, 2/3_ and *k*_ET, 2/4_

*k*_ET, 2/3_ and *k*_ET, 2/4_ were calculated using the equations below:

$$\Phi_{ET,2/3+2/4}=\frac{k_{ET,2/3}+k_{ET,2/4}}{1/\tau_{1/2}{+k}_{ET,2/3}+k_{ET,2/4}} {(\tau}_{1/2}=2.99 ns)$$

$$\Phi_{ET,2/3+2/4}=1-\frac{I_{Cou343}}{I_{Cou343(100/10/6/0)}}\times(1-\Phi_{ET,2/3(100/10/6)}) \Phi_{ET,2/3(100/10/6)}=0.925$$

Therefore, to obtain the *k_ET_* values, two additional ternary systems of **1**/**2**/**3** and **1**/**2**/**4** were investigated, as shown in Figure S26. The excitation wavelength was 425 nm. The calculated *Φ_ET_* values are listed in Table S10.

*
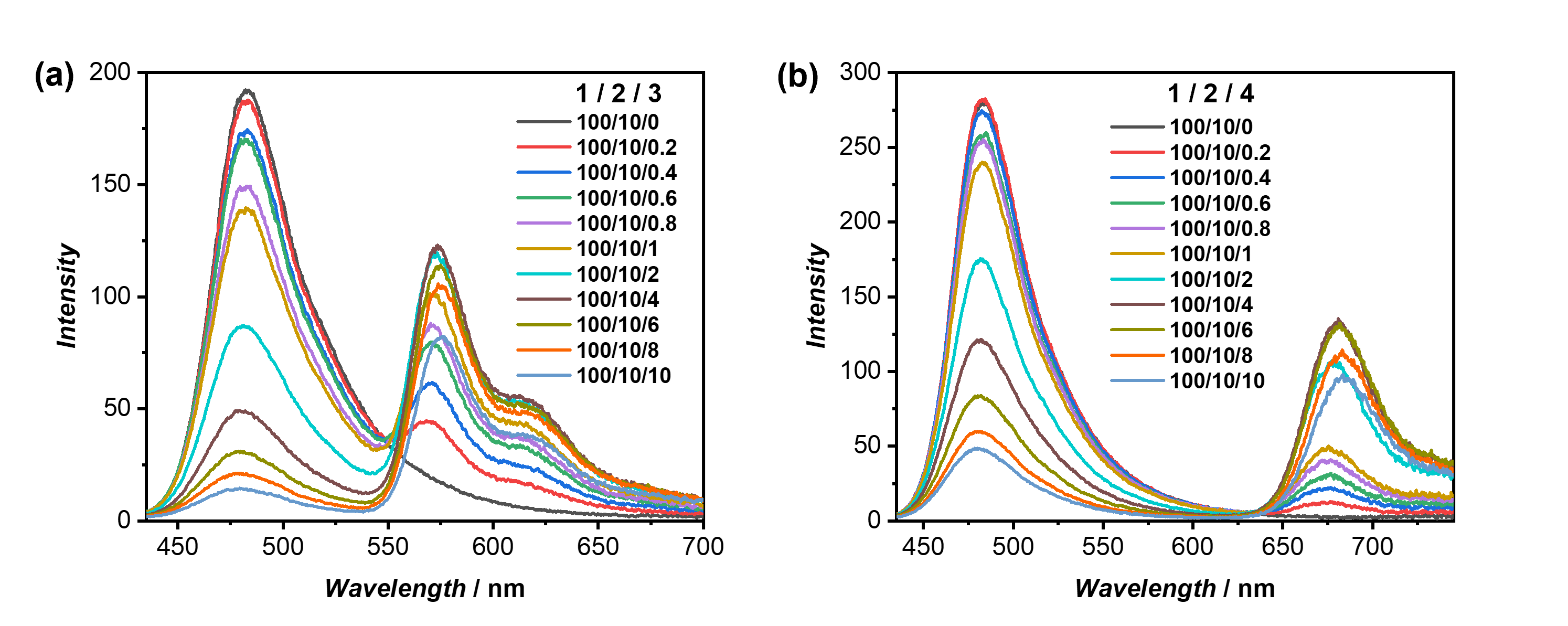
*

Figure S26 Fluorescence spectra of DPA-CP-PEG/Cou343-CP = 100/10 with different concentrations of Cy3-CP (a) and Cy5-CP (b) (*λ*_ex_ = 425 nm).

Table S10 Calculated *Φ*_ET_ values of the ternary systems of **1**/**2**/**3** and **1**/**2**/**4** excited at 425 nm.

|  | **1/2/3** | **1/2/4** |
| --- | --- | --- |
| Ratio | *Φ*_ET_ | |
| 100/10/0 | - | - |
| 100/10/0.4 | 0.097 | 0.026 |
| 100/10/0.6 | 0.117 | 0.078 |
| 100/10/0.8 | 0.226 | 0.090 |
| 100/10/1 | 0.274 | 0.148 |
| 100/10/2 | 0.544 | 0.380 |
| 100/10/4 | 0.744 | 0.571 |
| 100/10/6 | 0.839 | 0.703 |
| 100/10/8 | 0.891 | 0.785 |
| 100/10/10 | 0.926 | 0.828 |

Taking **1/2/3/4** = **100/10/6/6** as an instance (using the data from Table S9 and Table S10):

$$\frac{k_{ET,2/3}}{k_{ET,2/4}}=\frac{\Phi_{ET,2/3}}{\Phi_{ET,2/4}}\times\frac{1-\Phi_{ET,2/4}}{1-\Phi_{ET,2/3}}=\frac{0.839}{0.703}\times\frac{1-0.703}{1-0.839}=2.218({{1000}/{100}}/{60})$$

$$\Phi_{ET,2/3+2/4}=\frac{k_{ET,2/3}+k_{ET,2/4}}{1/\tau_{1/2}{+k}_{ET,2/3}+k_{ET,2/4}}=0.962 {(\tau}_{1/2}=2.99 ns)$$

Which gives *k*_ET, 2/3_ and *k*_ET, 2/4_ as 5.79×10^9^ s^-1^_,_ and 2.61×10^9^ s^-1^, respectively.

*c. Calculation of k*_ET, 3/4_

*k_ET,3/4_* was calculated using the equation below:

$$\Phi_{ET,3/4}=\frac{k_{ET,3/4}}{1/\tau_{1/2/3}{+k}_{ET,3/4}} {(\tau}_{1/2/3}=1.41 ns)$$

Table S11 Calculated *k*_ET_ values of the **1**/**2**/**3/4** quaternary system.

| Ratio | *k*_ET, 1/2_ / s^-1^ | *k*_ET, 1/3_ / s^-1^ | *k*_ET, 1/4_ / s^-1^ | *k*_ET, 2/3_ / s^-1^ | *k*_ET, 2/4_ / s^-1^ | *k*_ET, 3/4_ / s^-1^ |
| --- | --- | --- | --- | --- | --- | --- |
| 100/10/6/0.4 | 2.85×10^9^ | 2.40×10^9^ | 4.44×10^7^ | 3.04×10^9^ | 7.70×10^8^ | 1.41×10^8^ |
| 100/10/6/0.6 | 2.89×10^9^ | 2.43×10^9^ | 6.39×10^7^ | 2.36×10^9^ | 1.50×10^9^ | 2.57×10^8^ |
| 100/10/6/0.8 | 2.92×10^9^ | 2.46×10^9^ | 1.14×10^8^ | 3.00×10^9^ | 1.02×10^9^ | 3.85×10^8^ |
| 100/10/6/1 | 2.97×10^9^ | 2.50×10^9^ | 1.34×10^8^ | 2.90×10^9^ | 1.34×10^9^ | 5.48×10^8^ |
| 100/10/6/2 | 3.06×10^9^ | 2.57×10^9^ | 3.02×10^8^ | 3.21×10^9^ | 1.65×10^9^ | 1.42×10^9^ |
| 100/10/6/4 | 3.33×10^9^ | 2.80×10^9^ | 7.43×10^8^ | 4.43×10^9^ | 2.03×10^9^ | 4.08×10^9^ |
| 100/10/6/6 | 3.66×10^9^ | 3.08×10^9^ | 1.22×10^9^ | 5.79×10^9^ | 2.61×10^9^ | 8.35×10^9^ |

*d. Calculation of the Ф_ET_ values of the quaternary system* ***1/2/3/4***

$$\Phi_{ET,1/2}=\frac{k_{ET,1/2}}{1/\tau_{1}{+k}_{ET,1/2}+k_{ET,1/3}+k_{ET,1/4}}$$

$$\Phi_{ET,1/3}=\frac{k_{ET,1/3}}{1/\tau_{1}{+k}_{ET,1/2}+k_{ET,1/3}+k_{ET,1/4}}$$

$$\Phi_{ET,1/4}=\frac{k_{ET,1/4}}{1/\tau_{1}{+k}_{ET,1/2}+k_{ET,1/3}+k_{ET,1/4}}$$

$$\Phi_{ET,2/3}=\frac{k_{ET,2/3}}{1/\tau_{1/2}{+k}_{ET,2/3}+k_{ET,2/4}}$$

$$\Phi_{ET,2/4}=\frac{k_{ET,2/4}}{1/\tau_{1/2}{+k}_{ET,2/3}+k_{ET,2/4}}$$

$$\Phi_{ET,1-2-3-4}=\Phi_{ET,1/2}\times\Phi_{ET,2/3}\times\Phi_{ET,3/4}$$

$${\Phi_{ET,1-2-4}=\Phi}_{ET,1/2}\times\Phi_{ET,2/4}$$

$${\Phi_{ET,1-3-4}=\Phi}_{ET,1/3}\times\Phi_{ET,3/4}$$

$$\Phi_{ET,overall}=\Phi_{ET,1/2}\times\Phi_{ET,2/3}\times\Phi_{ET,3/4}+\Phi_{ET,1/2}\times\Phi_{ET,2/4}+\Phi_{ET,1/3}\times\Phi_{ET,3/4}+\Phi_{ET,1/4}$$

Table S12 Calculated *Ф*_ET_ values of the **1**/**2**/**3/4** quaternary system.

| Ratio | *Φ*_ET,1/2_ | *Φ*_ET,1/3_ | *Φ*_ET,1/4_ | *Φ*_ET,2/3_ | *Φ*_ET,2/4_ | *Φ*_ET,3/4_ | *Φ*_ET,total_ | *Φ*_ET,1-2-3-4_ | *Φ*_ET,1-2-4_ | *Φ*_ET,1-3-4_ | *Φ*_ET,1-4_ |
| --- | --- | --- | --- | --- | --- | --- | --- | --- | --- | --- | --- |
| 1000/100/60/0 | 0.521 | 0.438 | - | 0.925 | - | - | - | - | - | - | - |
| 100/10/6/0.4 | 0.513 | 0.432 | 0.011 | 0.562 | 0.359 | 0.227 | 0.361 | 0.066 | 0.184 | 0.099 | 0.011 |
| 100/10/6/0.6 | 0.509 | 0.428 | 0.020 | 0.689 | 0.234 | 0.305 | 0.380 | 0.109 | 0.119 | 0.133 | 0.020 |
| 100/10/6/0.8 | 0.507 | 0.427 | 0.023 | 0.634 | 0.293 | 0.385 | 0.456 | 0.122 | 0.149 | 0.162 | 0.023 |
| 100/10/6/1 | 0.494 | 0.416 | 0.049 | 0.618 | 0.318 | 0.619 | 0.653 | 0.190 | 0.157 | 0.258 | 0.049 |
| 100/10/6/2 | 0.467 | 0.393 | 0.104 | 0.652 | 0.299 | 0.823 | 0.816 | 0.250 | 0.140 | 0.323 | 0.104 |
| 100/10/6/4 | 0.446 | 0.375 | 0.148 | 0.663 | 0.299 | 0.905 | 0.892 | 0.269 | 0.133 | 0.342 | 0.148 |

**S6. Quinary LHS with Four-Step Sequential Energy Transfer**


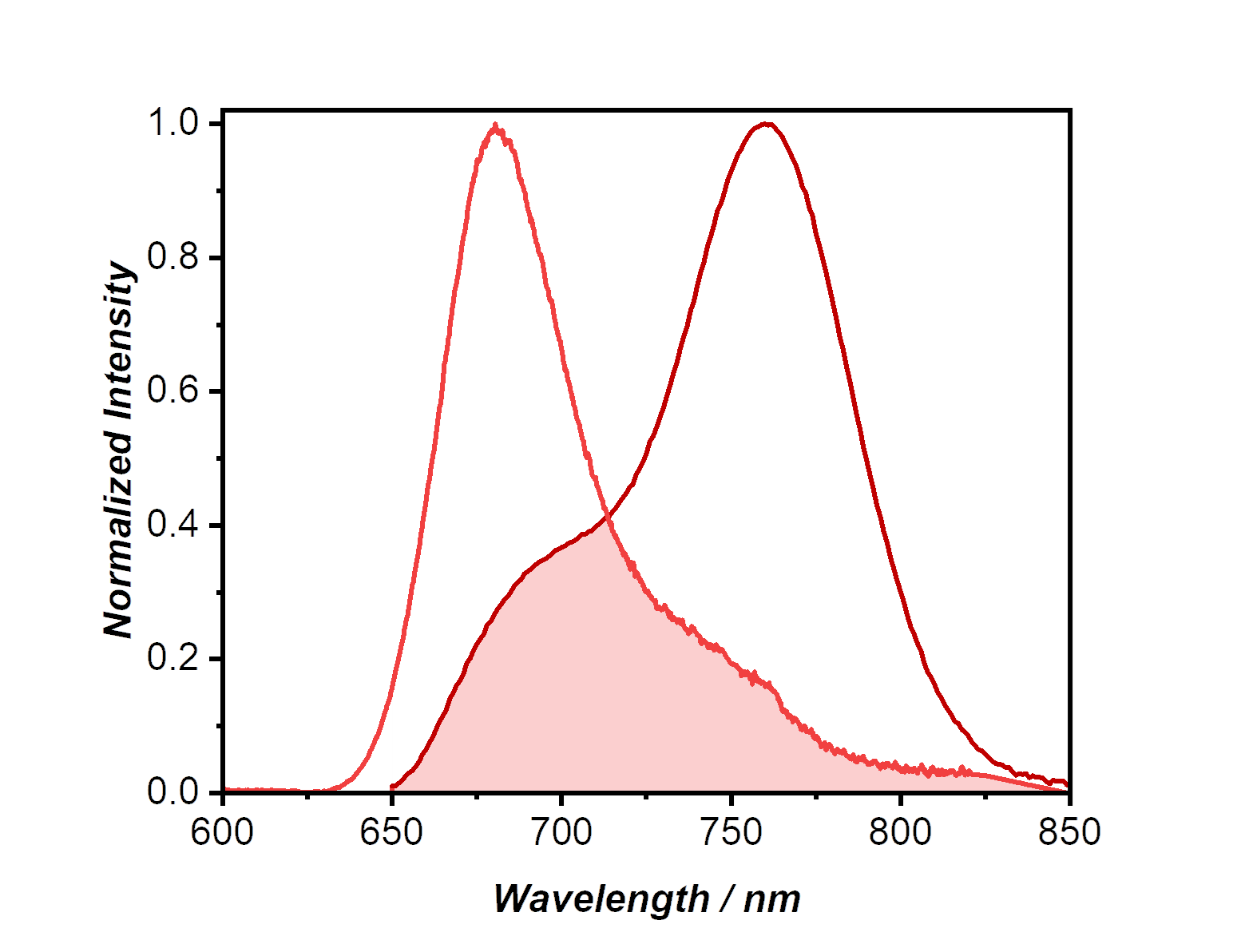


Figure S27 Spectral overlap of the normalized fluorescence spectrum of **4** (red) and absorption spectrum of **5** (dark red).
